# Dynamic nomogram integrating Gd-EOB-DTPA enhanced MRI semantic features, nutritional-inflammatory indices, and early treatment response to predict long-term survival in unresectable HCC treated with interventional, targeted, and immunotherapy: a multicenter retrospective study

**DOI:** 10.3389/fimmu.2026.1848104

**Published:** 2026-07-01

**Authors:** Rui-rui Sun, Hao-yang Tan, Yi-kang Wang, Yan-han Liu, Xue Zhou, Hua-guo Feng, Hai-ling Liu, Zuo-jin Liu

**Affiliations:** 1 Department of Hepatobiliary Surgery, The Second Affiliated Hospital of Chongqing Medical University, Chongqing, China; 2 Department of Radiology, The Chongqing University Jiangjin Hospital, School of Medicine, Chongqing University, Chongqing, China; 3 Department of Hepatobiliary Surgery, Chongqing Red Cross Hospital, Chongqing, China; 4 Chongqing Municipal Health Commission Key Laboratory of Precision Diagnosis and Treatment of Liver Cirrhosis and Complications, Chongqing, China

**Keywords:** hepatic arterial infusion chemotherapy, hepatocellular carcinoma, immunotherapy, magnetic resonance imaging, prognostic model

## Abstract

**Objective:**

This study developed and validated a dynamic prediction model combining MRI features, clinical factors, and early treatment response to noninvasively predict long-term survival in unresectable hepatocellular carcinoma patients undergoing triple therapy (hepatic arterial infusion chemotherapy, targeted therapy, and immunotherapy).

**Methods:**

This retrospective study enrolled 223 patients from two centers, with 127 in the training set and 96 in the external set, evaluating imaging features and constructing a dynamic nomogram to predict 1-year and 2-year survival. Model performance was evaluated via bootstrap resampling and external validation. Decision curve analysis and SHapley Additive exPlanations (SHAP) analysis were employed to assess clinical utility and model interpretability. An R/Shiny application was used for online deployment.

**Results:**

An imaging score was created based on five semantic features that suggest poor prognosis. The dynamic model, incorporating five semantic imaging features indicating poor prognosis, showed good discrimination, calibration, and clinical utility, especially when combining baseline and early treatment response. The predictive performance of the dynamic nomogram was numerically improved to that of BCLC staging and the traditional liver function grading system. SHAP analysis highlighted the imaging score as the most important predictor. The dynamic nomogram effectively stratified patients into low-, medium-, and high-risk groups, with significantly different survival outcomes.

**Conclusion:**

A dynamic nomogram model that integrates baseline features and therapeutic response provides a noninvasive and effective tool for predicting prognosis and stratifying risk in uHCC patients receiving triple therapy.

## 1 Introduction

Hepatocellular carcinoma (HCC) ranks as the sixth most common cancer and the third leading cause of cancer-related mortality worldwide (1). Most patients are diagnosed at advanced stages, where curative treatment options are limited (2). The combination of targeted therapy and immunotherapy has become the standard first-line therapy for unresectable hepatocellular carcinoma (uHCC) (3, 4). This regimen has demonstrated promising efficacy when combined with hepatic arterial infusion chemotherapy (HAIC), constituting a new triple therapy approach (HAIC+targeted therapy+immunotherapy) (5, 6). Despite these advancements, the median overall survival (OS) with combination therapy remains less than 24 months (7), and the objective response rate (ORR) is approximately 55.6% (8). The significant variability in OS and ORR across studies highlights the need for reliable predictive biomarkers to stratify patients and assist clinical decision−making effectively (9–11). To facilitate tailored prognostication, several prognostic models have been developed for uHCC patients receiving triple therapy, utilizing clinical features (12, 13), histopathological images (14), or radiomics (15, 16). Recently, studies have explored radiomics-based or multimodal deep learning models. Xu et al. created a CT radiomics and multimodal fusion system predicting treatment response and prognosis in large multicenter cohorts, with an AUC of 0.883 (17, 18).These methods often depend on complex imaging features and deep learning, limiting their generalizability. Developing a simple, clinically accessible prognostic tool based on conventional imaging features is essential for wider adoption of precision medicine.

Gadolinium ethoxybenzyl diethylenetriamine pentaacetic acid (Gd-EOB-DTPA) enhanced magnetic resonance imaging (MRI) is a valuable tool for the non-invasive preoperative diagnosis and prognostic assessment of HCC (19). Scoring models based on this imaging modality have been shown to effectively predict high-risk pathological features and early recurrence (20). Currently, there is no simple model that integrates Gd-EOB-DTPA-enhanced MRI semantic features, clinical data, and early treatment response to predict prognosis for uHCC patients undergoing triple therapy.

Therefore, this study aimed to develop and externally validate a straightforward dynamic prediction model that combines Gd-EOB-DTPA-enhanced MRI semantic features with baseline clinical factors and early treatment response features to predict long-term survival in uHCC patients receiving triple therapy.

## 2 Materials and methods

### 2.1 Patients

This retrospective study consecutively enrolled uHCC patients who received triple therapy at both centers from January 2021 through October 2024. The study was conducted in accordance with the Declaration of Helsinki, received Institutional Review Board approval (KY20240812–001 and 2026EC043), and was registered in the Chinese Clinical Trial Registry (ChiCTR2500095127). The requirement for informed consent was waived due to the use of de-identified clinical data. The research process and sample size calculation followed the updated TRIPOD and STROBE guidelines (21).

The inclusion criteria were as follows: (1) age≥18 years; (2) pathologically or clinically confirmed HCC; (3) Eastern Cooperative Oncology Group (ECOG) performance status of 0-1, Child-Pugh class A or B; (4) unresectable HCC confirmed by multidisciplinary consultation; (5) Gd-EOB-DTPA-enhanced MRI performed within one week before treatment initiation; (6) Follow up period exceeding 3 months; (7) first-line treatment with at least two cycles of triple therapy; and (8) complete clinical data and medical history. Our inclusion criteria required patients to complete at least 2 cycles of triple therapy and to have more than 3 months of follow-up. Thus, the model primarily applies to patients who tolerate and complete the induction phase.

The exclusion criteria were: (1) had previously received systemic treatment for HCC (including TKIs, ICIs, or chemotherapy); (2) distant metastasis (e.g., brain or lung); (3) severe immune, cardiac, pulmonary, or renal comorbidities; and (4) incomplete medical records.

### 2.2 Triple therapy

All patients received mFOLFOX-HAIC (22) combined with first-line systemic therapy (Apatinib and Camrelizumab) according to guideline recommendations (23). The mFOLFOX-HAIC regimen consisted of oxaliplatin (85 mg/m² infused over 3 hours on day 1), leucovorin (400 mg/m² infused over 2 hours), an arterial bolus of 5-fluorouracil (400 mg/m²), followed by continuous arterial infusion of 5-fluorouracil (2500 mg/m² over 46 hours). Apatinib was administered orally at a fixed dose of 250 mg once daily, and Camrelizumab was given intravenously at 200 mg. Both HAIC and Camrelizumab were administered in 3-week cycles. Early tumor response was assessed using imaging after two cycles (approximately 6 weeks), and subsequent treatment decisions (such as surgical resection, continuation of local therapy, or second-line drug treatment) were made through multidisciplinary discussion.

### 2.3 Data collection and definition

Data were collected from the electronic medical record system. Laboratory and imaging data were obtained within 24 hours before the initial HAIC session. Patients were followed up monthly via outpatient visits or telephone calls until October 31, 2025.

Primary outcomes were early tumor response, ORR, disease control rate (DCR), OS, 1-year survival rate, and 2-year survival rate. For the baseline model that includes only pre−treatment variables, OS was calculated from the date of initial diagnosis. For the integrated model, which incorporates early treatment response, revised OS was calculated from the date of the first response evaluation after two treatment cycles (approximately 1.5 months) to avoid immortal time bias; thus, all patients in the integrated model had survived to that time point. The early tumor response following two treatment cycles was assessed using the modified Response Evaluation Criteria in Solid Tumors (mRECIST) (24). ORR and DCR were defined according to mRECIST criteria. Alpha-fetoprotein (AFP) response was defined as a reduction of >50% in AFP levels following treatment (25).

Inflammatory biomarkers were calculated from complete blood count and albumin measurements using the following formulas:

Platelet-to-lymphocyte ratio (PLR) = platelet count (×10^9^/L)/lymphocyte count (×10^9^/L);
Neutrophil-to-lymphocyte ratio (NLR) = neutrophil count (×10^9^/L)/lymphocyte count (×10^9^/L);
Systemic immune inflammation index (SII) = platelet count (×10^9^/L) × neutrophil count (×10^9^/L)/lymphocyte count (×10^9^/L);
Prognostic nutritional index (PNI) = albumin (g/L) + 5 × lymphocyte count (×10^9^/L);
C-reactive protein-to-albumin ratio (CAR)=C-reactive protein(mg/L)/albumin(g/L).

### 2.4 Image feature extraction

Gd-EOB-DTPA-enhanced MRI was performed using 3.0-T scanners at both centers. Detailed acquisition parameters are provided in **Supplementary Appendix S1** and **Supplementary Table 1**.

Image analysis was performed independently by two experienced radiologists (Xue Zhou, 10 years of abdominal MRI experience; Yi Wang, 8 years of abdominal MRI experience) who were blinded to clinical data. Detailed definitions of imaging features are provided in **Supplementary Table 2**. Randomly selected 100 cases (50 from each center) and evaluated inter-observer agreement for imaging features using Cohen’s Kappa analysis (26). Features with a kappa <0.75 were re-evaluated by consensus or consultation with a senior radiologist (15 years of experience). The final imaging features used for modeling were based on the consensus labels. For patients with multi-focal HCC, a feature was recorded as present if any lesion exhibited that feature, following LI-RADS v2018 guidelines (27).

### 2.5 Statistical analysis

#### 2.5.1 Model development and nomogram construction

The imaging score was derived from a Cox regression with an optimal cutoff. LASSO regression (lambda.1se) identified key clinical features (28), which were then entered into a multivariate Cox regression to determine independent risk factors. Multicollinearity was assessed using the variance inflation factor (VIF) (29). A dynamic nomogram (baseline and integrated models) that incorporates baseline features and therapeutic response was constructed to visualize 1-year and 2-year survival probabilities. To further assess the potential impact of immortal time bias, we constructed a time−dependent Cox regression model for sensitivity analysis. ORR and AFP response were treated as time−varying covariates, set to 0 before the first response evaluation (1.5 months) and to the observed values thereafter. The C−index and hazard ratios of the time−dependent model were compared with those of the original integrated model to evaluate the magnitude of immortal time bias. The baseline model provides pre−treatment risk stratification to inform clinical decision−making, and the integrated model, applied after two cycles, offers updated prognostic information that may guide treatment continuation.

#### 2.5.2 Model validation and assessment

Internal validation was performed using 500 bootstrap resamples, and external validation was conducted in an independent cohort. Model performance was evaluated using discrimination [Harrell’s concordance index (C-index) and time-dependent area under curve (AUC)], improvement [net reclassification index (NRI) and integrated discrimination improvement (IDI)], calibration [assessed by Hosmer−Lemeshow test, calibration slope, calibration intercept, Brier score, and integrated Brier score (IBS)]) (30), and clinical utility [decision curve analysis (DCA)]. The binary risk threshold for calculating sensitivity and specificity was defined as the risk score that maximized the Youden index in the training set. Brier scores were calculated at specific time points (1-year and 2-year), and IBS was computed as the average Brier score from time 0 to the maximum follow−up. To assess the generalizability of the model across different clinical subgroups, pre−specified subgroup analyses were performed in the training and external validation cohorts based on age (<60 vs ≥60 years), gender, BMI (<24 vs ≥24 kg/m²), cirrhosis (present vs absent), PS score (0 vs 1), Child−Pugh class (A vs B), BCLC stage (0 vs 1), ALBI grade (1 vs 2), and AFP level (negative vs positive). To assess the impact of subsequent treatments on model performance, a censoring sensitivity analysis was conducted. Risk stratification was performed using the trisection classification method, and survival curves were compared between risk groups.

#### 2.5.3 Model interpretability

SHapley Additive exPlanations (SHAP) analysis was applied to interpret Cox model outputs and rank feature importance (31). An R/Shiny application (https://www.shinyapps.io/) was developed for online deployment of the dynamic nomogram.

#### 2.5.4 Data analysis

Statistical analyses were performed using R software (version 4.5.1). Continuous data are presented as mean ± standard deviation or median with interquartile range (25th to 75th percentile), based on their distribution. Categorical data are presented as frequencies (percentages). Multiple imputation (mice package in R, 5 imputations) was used for missing continuous variables. For categorical variables, mode imputation was applied. Baseline characteristics were compared using Student’s t-test, Mann-Whitney U test, Chi-square test, or Fisher’s exact test, as appropriate. The maximally selected rank statistics method was used to determine the optimal cut-off points for continuous variables (32). Restrictive cubic splines (RCS) were used to detect non-linear relationships among the variables ultimately included in the model, and the continuous variables before binary classification were re-fitted to the COX model for sensitivity analysis. Differences in OS between groups were assessed using the log-rank test. Differences between AUC values were compared using the method described by Delong et al. (33). A two-tailed P-value <0.05 was considered statistically significant.

## 3 Results

### 3.1 Patient characteristics

**Figure 1:**
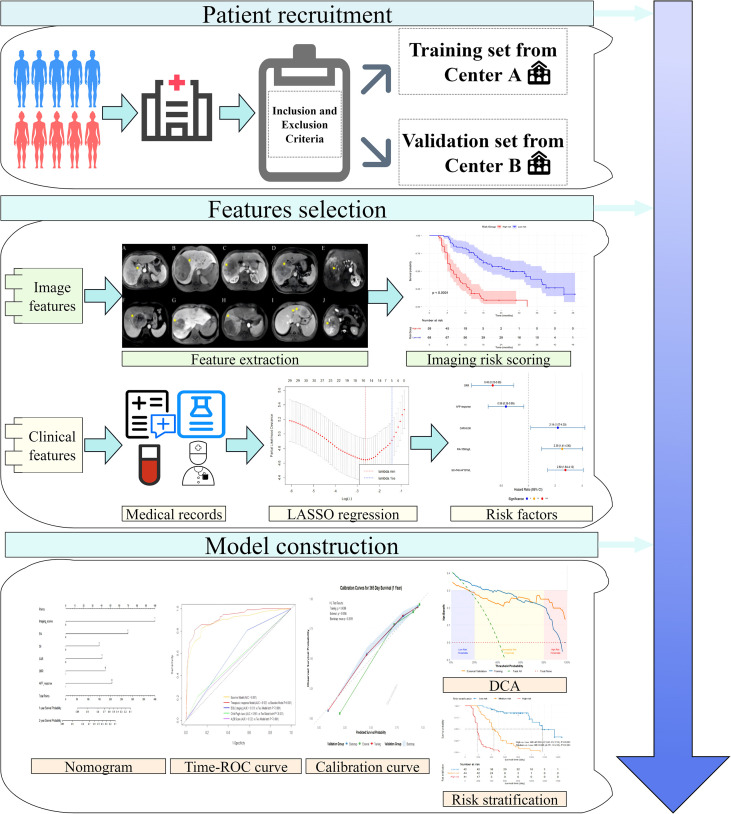
The schematic outline of the study.

A total of 271 uHCC patients who received triple therapy were initially enrolled from two centers. After eligibility screening, 223 patients were included in the final analysis: 127 from Center 1 (training set) and 96 from Center 2 (external validation set) (**Figure 1**). Baseline characteristics were well balanced between the two sets (**Supplementary Table 3**). After two treatment cycles, the early tumor response was evaluated for all patients (**Supplementary Figure 1**). The ORR was 39.5% (88/223), the DCR was 94.6% (211/223), and the AFP response rate was 51.1% (114/223).

All patients were successfully followed up (loss of follow-up rate was 0), with a median follow-up time of 21.5 months (range: 16.5-28.8). The median follow-up time in the training set was longer than in the external validation set [24.6 (17.7-29.5) vs. 20.4 (16.5-24.4); P = 0.036]. By the end of the follow-up period, 64 patients (28.7%) upgraded to second-line drug treatment, 21 patients (9.4%) had undergone surgical resection, 201 (90.1%) had experienced tumor progression, 64 (28.7%) had developed distant metastases, and 157 (70.4%) had died. No significant differences in short-term or long-term efficacy endpoints were observed between the training and validation sets (**Supplementary Figure 2**).

Based on Riley’s method (34), 6 predictor variables, a 1-year mortality rate of 38.6%, a 1-year time point, a median follow-up of 25.9 months, and an assumed Nagelkerke’s R² of 0.3, the required sample size was calculated to be 177 patients (2-year’s sample size was 182).

### 3.2 Image feature extraction

**Figure 2:**
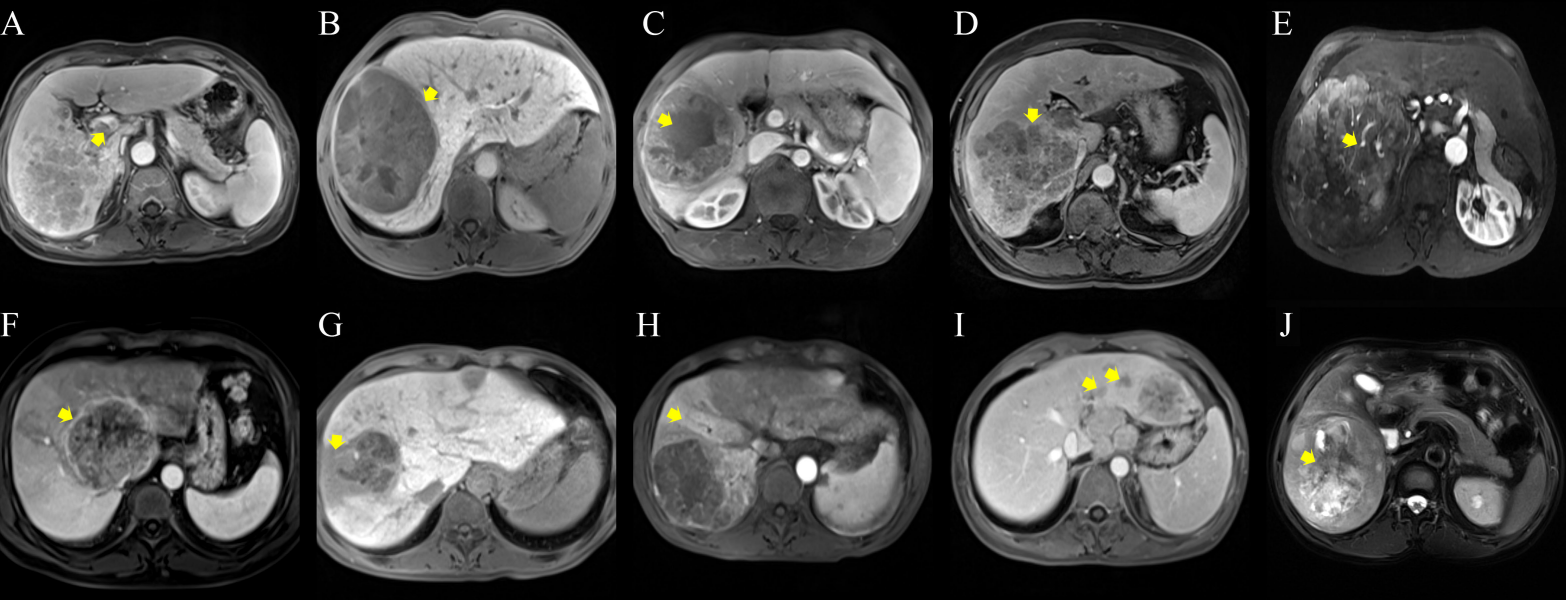
Extracted imaging features from Gd-EOB-DTPA-enhanced MRI: **(A)** Vascular invasion in portal venous phase, **(B)** Complete capsule in hepatobiliary phase, **(C)** Intratumoral necrosis in T2WI, **(D)** Irregular morphology in portal venous phase, **(E)** Intratumoral arteries in arterial phase, **(F)** Rim enhancement in arterial phase, **(G)** Peritumoral low signal in hepatobiliary phase, **(H)** Peritumoral enhancement in arterial phase, **(I)** Satellite lesions in portal venous phase, and **(J)** Mosaic sign in T2WI. Yellow arrows indicate imaging features.

**Figure 3:**
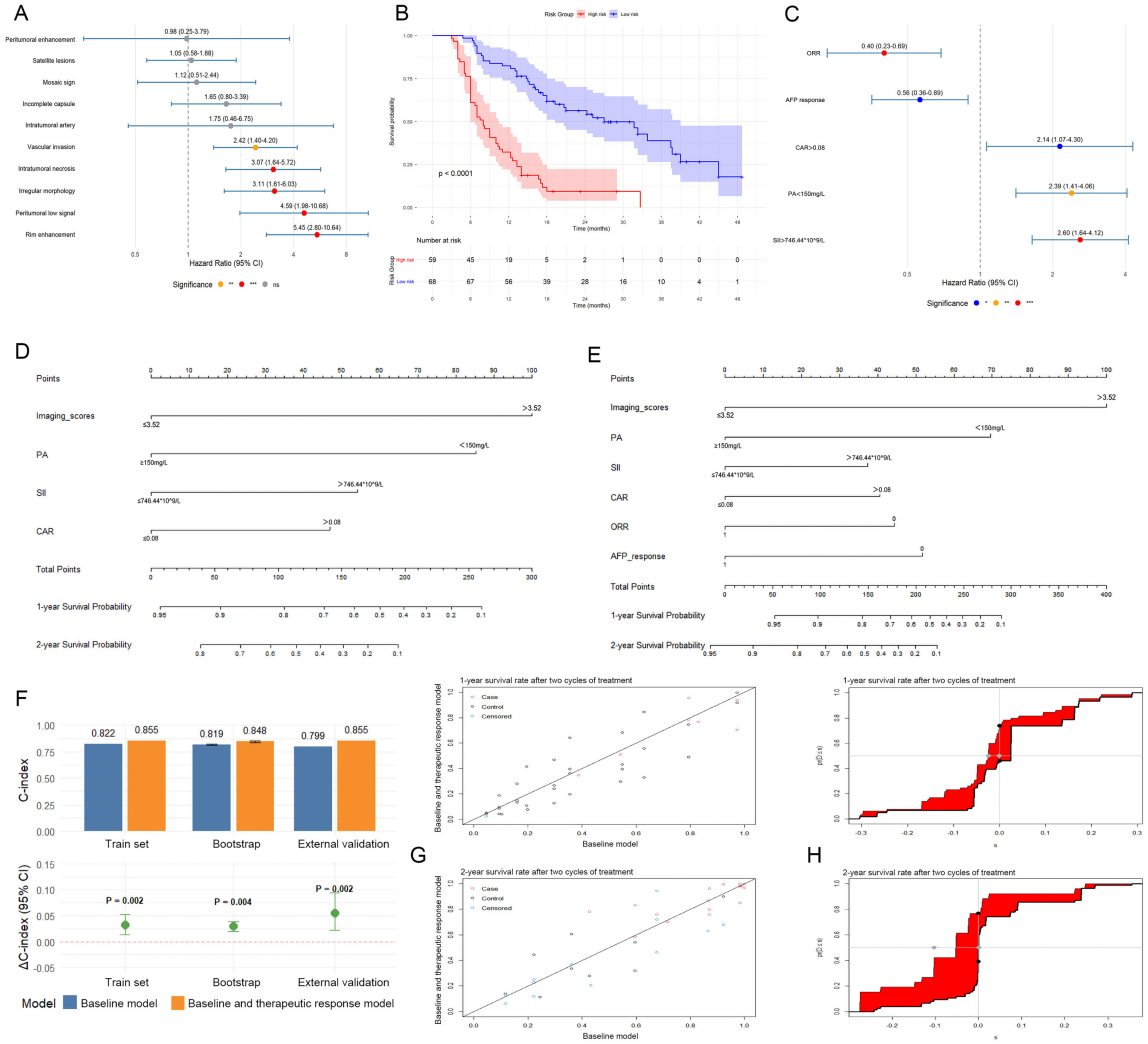
Features selection and nomogram construction. **(A)** Multivariate Cox analysis of imaging features forest plot; **(B)** Overall survival curve of risk stratification in imaging score (the optimal cut-off value of 3.52); **(C)** Multivariate Cox analysis of clinical features (after LASSO regression) forest plot; **(D)** Construction of the baseline nomogram model including baseline features; **(E)** Construction of the integrated nomogram model including baseline features and initial therapeutic response; **(F)** C-index comparison of both models; **(G)** Scatter diagram of continuous net reclassification index for 1-year and 2-year survival rates (more ‘Case’ above the diagonal or more ‘Control’ below the diagonal indicated that the integrated model improved over the baseline model); **(H)** Integrated discrimination improvement classification chart for 1-year and 2-year survival rates (a large red area indicated the integrated model improved over the baseline model). HR, hazard ratio; PA, prealbumin; SII, systemic Immune Inflammation index; CAR, C-reactive protein to albumin ratio; ORR, objective response rate; AFP, alpha-fetoprotein.

Cohen’s kappa coefficients for imaging features ranged from 0.905 to 0.949 (mean 0.923), indicating strong interobserver agreement (**Supplementary Table 4**). Representative examples are shown in **Figures 2A–J**, and their frequencies are detailed in **Table 1**. Multivariate Cox regression identified five independent risk factors for OS: vascular invasion [hazard ratio(HR)=2.42 (1.40–4.20), P = 0.002], intratumoral necrosis [HR = 3.07 (1.64–5.72), P<0.001], irregular morphology [HR = 3.11 (1.61–6.03), P<0.001], rim enhancement [HR = 5.45 (2.80–10.64), P<0.001], and peritumoral low signal in the hepatobiliary phase [HR = 4.59 (1.98–10.68), P<0.001] (**Figure 3A**).

**Table 1:** Imaging features of all patients included in the study.

Imaging features	Total cohort (n=223)	Training cohort (n=127)	Validation cohort (n=96)	Statistic	*P value
Vascular invasion, n (%)	151 (67.7%)	82 (64.6%)	69 (71.9%)	χ²=1.022	0.312
Incomplete capsule, n (%)	108 (48.4%)	63 (49.6%)	45 (46.9%)	χ²=0.072	0.788
Intratumoral necrosis, n (%)	65 (29.1%)	40 (31.5%)	25 (26.0%)	χ²=0.546	0.460
Irregular morphology, n (%)	119 (53.4%)	70 (55.1%)	49 (51.0%)	χ²=0.220	0.639
Intratumoral arteries, n (%)	97 (43.5%)	56 (44.1%)	41 (42.7%)	χ²=0.005	0.944
Rim enhancement, n (%)	135 (60.5%)	83 (65.4%)	52 (54.2%)	χ²=2.415	0.120
Peritumoral low signal, n (%)	83 (37.2%)	50 (39.4%)	33 (34.4%)	χ²=0.390	0.533
Peritumoral enhancement, n (%)	96 (43.0%)	53 (41.7%)	43 (44.8%)	χ²=0.103	0.749
Satellite lesions, n (%)	47 (21.1%)	26 (20.5%)	21 (21.9%)	χ²=0.008	0.929
Mosaic sign, n (%)	78 (35.0%)	45 (35.4%)	33 (34.4%)	χ²=0.001	0.982

* Comparison between training set and validation set.

The imaging score was calculated as: 0.885 × vascular invasion (yes=1, no=0) + 1.120 × intratumoral necrosis + 1.135 × irregular morphology + 1.696 × rim enhancement + 1.525 × peritumoral low signal in hepatobiliary phase. The optimal cut-off value of 3.52 was used to define high-risk (>3.52) and low-risk (≤3.52) groups, which showed significantly different OS [HR = 4.76 (3.01–7.55), P<0.001; **Figure 3B**].

### 3.3 Clinical feature selection and nomogram construction

The optimal cut-off values for continuous clinical variables were determined using the maximally selected rank statistics method (**Supplementary Table 5**). LASSO regression analysis identified five key variables, which were subsequently included in the multivariate Cox analysis: prealbumin (PA) < 150 mg/L [HR = 2.39 (1.41–4.06), P = 0.001], SII >746.44 × 10^9^/L [HR = 2.60 (1.64–4.12), P<0.001], CAR > 0.08 [HR = 2.14 (1.07–4.30), P = 0.033], ORR [HR = 0.40 (0.23–0.69), P<0.001], and AFP response [HR = 0.56 (0.36–0.89), P = 0.015] (**Supplementary Figure 3**; **Figure 3C**). These six predictors—four baseline and two response-related—formed the dynamic prognostic nomogram (baseline model: **Figure 3D**; integrated model: **Figure 3E**). No missing data were present for these variables. Baseline model risk score formula: 1.2816 × PA (<150 mg/L as 1)+0.815 × SII (>746.44×10^9^/L as 1)+0.7047 × CAR (>0.08 as 1) +1.5026 × Imaging scores (>3.52 as 1). Baseline survival (all variables set to 0): 1-year survival probability was 0.955, and 2-year survival probability was 0.884. Integrated model risk scoring formula: lp = 1.1126 × PA + 0.5981 × SII + 0.6493 × CAR + 1.5997 × Imaging scores + (-0.7106) × ORR (response as 1) + (-0.827) × AFP response (response as 1). Baseline 1-year survival probability was 0.901, and 2-year survival probability was 0.736.

The comparison of key variables in the dynamic model between the training and validation sets is presented in the **Table 2**. There were statistical differences in the distributions of model key variables (PA<150 mg/L and CAR>0.08) between the two sets (P = 0.033 and P<0.001, respectively). The number of deaths in the training set was 91, yielding an events−per−variable ratio of 91/6 = 15.2, which is well above the recommended minimum of 10, indicating low risk of overfitting. The RCS results showed that no significant non−linear terms for any of the continuous variables (all P > 0.05). A sensitivity analysis was performed on the binary classification of continuous variables in the models (**Supplementary Figure 4**). The results showed no significant change in the model’s discriminability. VIF values ranged from 1.19 to 1.28, indicating no significant multicollinearity (**Supplementary Figure 5**).

**Table 2:** Comparison of key variables in the model between the training and validation sets.

Variable	Training (n=127)	External (n=96)	Statistic	P value
Death events	91 (71.7%)	66 (68.8%)	χ²=0.216	0.642
PA (<150 mg/L)	79 (62.2%)	46 (47.9%)	χ²=4.530	0.033*
SII (>746.44*10^9^/L)	52 (40.9%)	37 (38.5%)	χ²=0.130	0.718
CAR (>0.08)	99 (78%)	50 (52.1%)	χ²=16.500	<0.001*
Imaging scores (>3.52)	66 (52%)	38 (39.6%)	χ²=3.370	0.066
ORR	47 (37%)	41 (42.7%)	χ²=0.740	0.388
AFP response	67 (52.8%)	47 (49%)	χ²=0.320	0.572

PA, Prealbumin; SII, Systemic Immune Inflammation index; CAR, C-reactive protein to Albumin Ratio; ORR, objective response rate; AFP, Alpha-fetoprotein. * P < 0.05 indicates a statistically significant difference between the training and external validation cohorts.

### 3.4 Model validation and assessment

The baseline model’s C-index was 0.822, 0.819, and 0.799 across the training, bootstrap, and external validation sets. For the integrated model, the C-index was 0.855, 0.848, and 0.855, respectively (**Figure 3F**). At 1-year follow-up, the NRI was 0.567 (0.272–0.996), and the IDI was 0.050 (0.001–0.100), indicating that the integrated model significantly improved risk reclassification and prediction performance (P = 0.002; P = 0.040). At 2 years, the NRI was 0.653 (0.392–1.417), and the IDI was 0.100 (0.015–0.180), also significant (P = 0.012; P = 0.032) (**Supplementary Table 6**; **Figures 3G, H**).

**Figure 4:**
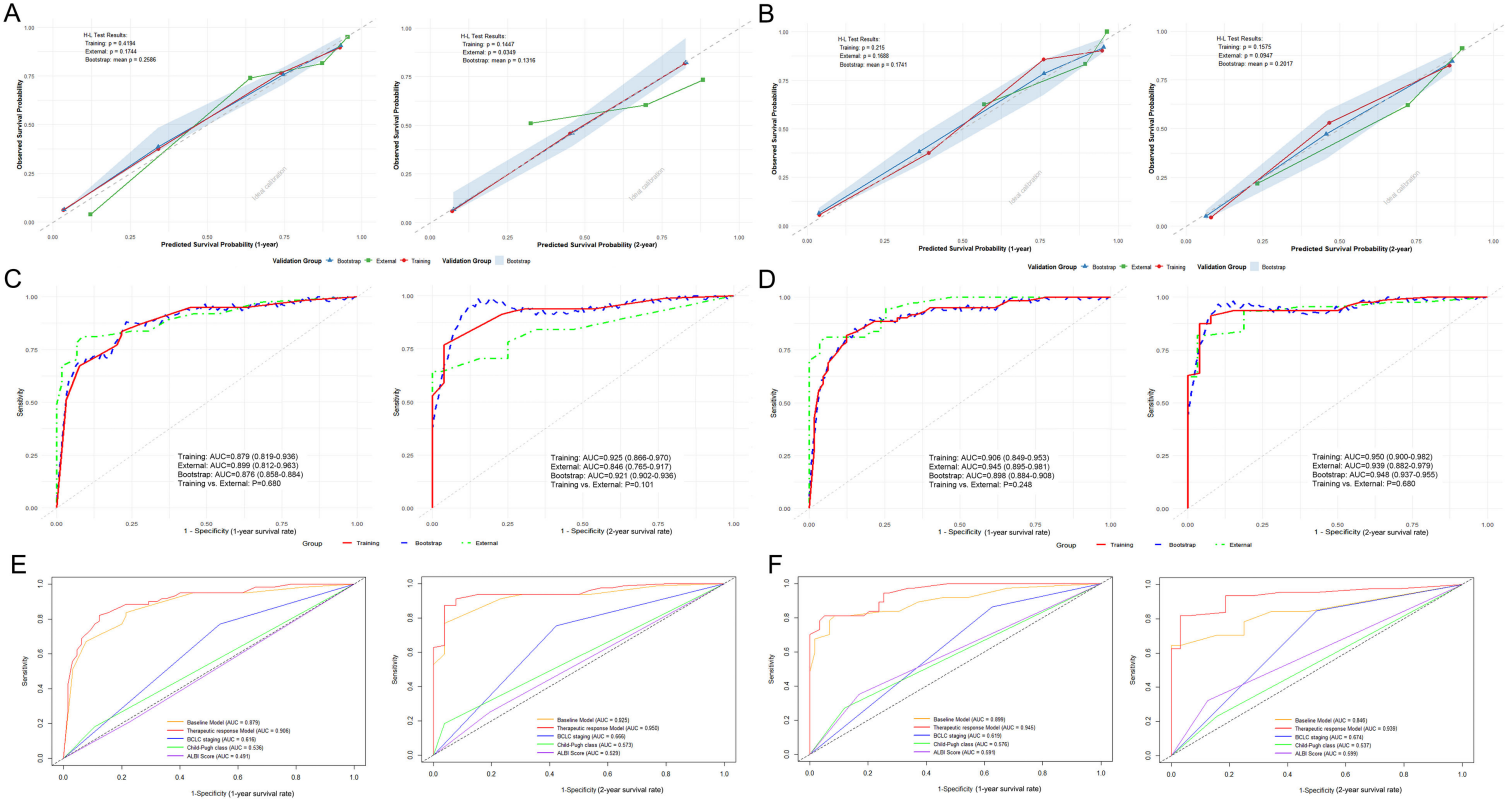
Validation and assessment of nomogram models. **(A)** Calibration curves of 1-year and 2-year survival rates for the baseline model; **(B)** Calibration curves of 1-year and 2-year survival rates for the integrated model; **(C)** The time-dependent receiver operating characteristic (ROC) curves for 1-year and 2-year survival rates in the baseline model; **(D)** The time-dependent ROC curves for 1-year and 2-year survival rates in the integrated model; **(E)** Comparison of the time-dependent ROC curves for 1-year and 2-year survival rates between the dynamic nomogram model and traditional indicators in the training cohort; **(F)** Comparison of the time-dependent ROC curves for 1-year and 2-year survival rates between the dynamic nomogram model and traditional indicators in the external validation cohort. AUC, area under the curve; BCLC, barcelona clinic liver cancer; ALBI, albumin-bilirubin ratio.

The calibration slope, intercept, Brier score, and IBS for the integrated model in the training and external validation sets are presented in **Supplementary Table 7**. The Brier score is below 0.15 in both the training and external validation sets, with IBS values of 0.082-0.113. These results collectively indicate that the integrated model maintains consistent calibration performance across both sets. In the baseline model, calibration curves for 1-year survival showed good agreement between predicted and observed outcomes in the training, bootstrap validation, and external validation sets. For 2-year survival, the nomogram also showed good calibration in both the training and bootstrap sets. However, there was a statistically significant difference between predicted and observed outcomes in the external validation set (P = 0.035) (**Figure 4A**). In the integrated model, calibration curves for 1-year and 2-year survival showed good agreement in the training, bootstrap validation, and external validation sets (**Figure 4B**).

Time-dependent AUCs for the baseline model were 0.879 (0.819-0.936) (training) and 0.899 (0.812-0.963) (external) at 1 year (P = 0.680). The optimal binary risk threshold was 0.412. Bootstrap AUC was 0.876 (0.858-0.884), indicating low overfitting (0.003). For 2-year survival, AUCs were 0.925 (0.866-0.970) (training) and 0.846 (0.765-0.917) (external) (P = 0.101). The optimal binary risk threshold was 0.523. Bootstrap AUC was 0.921 (0.902-0.936), again with low overfitting (0.004) (**Figure 4C**). For the integrated model, the AUCs were 0.906 (0.849-0.953) (training) and 0.945 (0.895-0.981) (external) at 1 year (P = 0.248). The optimal binary risk threshold was 0.386. Bootstrap AUC was 0.898 (0.884-0.908), indicating low overfitting (0.008). For 2-year survival, AUCs were 0.950 (0.900-0.982) (training) and 0.939 (0.882-0.979) (external) (P = 0.680). The optimal binary risk threshold was 0.481. Bootstrap AUC was 0.948 (0.937-0.955), again with low overfitting (0.002) (**Figure 4D**). The model’s performance metrics are summarized in **Table 3**.

**Table 3:** Performance of the dynamic nomogram model for predicting cumulative annual survival rate.

Cohort	AUC	Accuracy	Sensitivity	Specificity	PPV	NPV	P value*/ Optimism∇
Baseline model							
1-year survival rate							
Training cohort	0.879 (0.819-0.936)	0.809 (0.754-0.876)	0.836 (0.639-0.934)	0.785 (0.701-0.968)	0.785 (0.694-0.955)	0.836 (0.700-0.933)	
External cohort	0.899 (0.812-0.963)	0.875 (0.813-0.938)	0.811 (0.643-0.927)	0.915 (0.849-1.000)	0.857 (0.758-1.000)	0.885 (0.789-0.961)	0.680 (Z=-0.412)
Bootstrap validation	0.876 (0.858-0.884)	0.809 (0.794-0.818)	0.787 (0.656-0.869)	0.830 (0.723-0.923)	0.818 (0.747-0.889)	0.809 (0.741-0.855)	0.003
2-year survival rate							
Training cohort	0.925 (0.866-0.970)	0.824 (0.765-0.937)	0.780 (0.702-0.961)	0.962 (0.771-1.000)	0.985 (0.917-1.000)	0.581 (0.460-0.884)	
External cohort	0.846 (0.765-0.917)	0.786 (0.704-0.882)	0.654 (0.532-0.895)	1.000 (0.766-1.000)	1.000 (0.847-1.000)	0.640 (0.512-0.836)	0.101 (Z = 1.693)
Bootstrap validation	0.921 (0.902-0.936)	0.814 (0.787-0.870)	0.770 (0.732-0.902)	0.954 (0.769-0.962)	0.982 (0.925-0.985)	0.571 (0.532-0.714)	0.004
Baseline and therapeutic response integrated model							
1-year survival rate							
Training cohort	0.906 (0.849-0.953)	0.849 (0.792-0.913)	0.820 (0.712-0.946)	0.877 (0.750-0.967)	0.862 (0.754-0.963)	0.838 (0.758-0.950)	
External cohort	0.945 (0.895-0.981)	0.896 (0.813-0.958)	0.811 (0.694-0.990)	0.949 (0.734-1.000)	0.909 (0.667-1.000)	0.889 (0.814-0.992)	0.248 (Z=-1.154)
Bootstrap validation	0.898 (0.884-0.908)	0.837 (0.817-0.857)	0.819 (0.745-0.885)	0.853 (0.754-0.908)	0.842 (0.771-0.887)	0.836 (0.792-0.879)	0.008
2-year survival rate							
Training cohort	0.950 (0.900-0.982)	0.898 (0.853-0.972)	0.878 (0.817-0.975)	0.962 (0.857-1.000)	0.986 (0.948-1.000)	0.714 (0.589-0.927)	
External cohort	0.939 (0.882-0.979)	0.881 (0.828-0.952)	0.827 (0.759-0.979)	0.969 (0.787-1.000)	0.977 (0.875-1.000)	0.775 (0.671-0.964)	0.680 (Z = 0.412)
Bootstrap validation	0.948 (0.937-0.955)	0.904 (0.866-0.926)	0.892 (0.829-0.927)	0.944 (0.885-1.000)	0.981 (0.962-1.000)	0.738 (0.645-0.800)	0.002

AUC, Area Under the receiver operating characteristic Curve; PPV, Positive Predictive Value; NPV, Negative Predictive Value. Data in parentheses are 95 % confidence intervals. The optimal binary risk thresholds for predicting 1-year and 2-year mortality were determined by maximizing the Youden index in the training set. For the baseline model, the thresholds were 0.412 (1-year) and 0.523 (2-year); for the integrated model, the thresholds were 0.386 (1-year) and 0.481 (2-year).

*Comparison between training cohort and external validation cohort.

∇Optimism indicates the extent of overfitting between training cohort performance and Bootstrap validation performance.

Both the training set and the external validation set showed that the AUCs of the baseline and integrated models for 1-year and 2-year survival were numerically higher than those of BCLC staging, Child-Pugh classification, and ALBI score (**Figures 4E, F**). The detailed Delong results are shown in **Table 4**.

**Table 4:** The DeLong test of the models’ AUC values for 1-year and 2-year survival rates.

	AUC	Difference	Statistics	P value
Training set (1-year survival rate)				
Baseline model	0.879	/	/	/
*Vs. BCLC staging*	0.616	0.263	Z=5.114	<0.001
*Vs. Child-Pugh class*	0.536	0.343	Z=7.792	<0.001
*Vs. ALBI score*	0.491	0.388	Z=8.032	<0.001
Integrated model	0.906	/	/	/
*Vs. Baseline model*	0.879	0.027	Z = 0.649	0.517
*Vs. BCLC staging*	0.616	0.290	Z = 5.882	<0.001
*Vs. Child-Pugh class*	0.536	0.369	Z = 8.922	<0.001
*Vs. ALBI score*	0.491	0.415	Z = 9.020	<0.001
Training set (2-year survival rate)				
Baseline model	0.925	/	/	/
*Vs. BCLC staging*	0.666	0.257	Z=4.286	<0.001
*Vs. Child-Pugh class*	0.573	0.350	Z=9.034	<0.001
*Vs. ALBI score*	0.529	0.394	Z=7.504	<0.001
Integrated model	0.950	/	/	/
*Vs. Baseline model*	0.925	0.025	Z = 0.744	0.457
*Vs. BCLC staging*	0.666	0.282	Z = 4.844	<0.001
*Vs. Child-Pugh class*	0.573	0.374	Z = 10.441	<0.001
*Vs. ALBI score*	0.529	0.419	Z = 8.302	<0.001
External validation set (1-year survival rate)				
Baseline model	0.899	/	/	/
*Vs. BCLC staging*	0.619	0.280	Z=5.041	<0.001
*Vs. Child-Pugh class*	0.576	0.323	Z=5.821	<0.001
*Vs. ALBI score*	0.591	0.308	Z=5.246	<0.001
Integrated model	0.945	/	/	/
*Vs. Baseline model*	0.899	0.046	Z = 1.125	0.261
*Vs. BCLC staging*	0.619	0.326	Z = 6.889	<0.001
*Vs. Child-Pugh class*	0.576	0.369	Z = 7.806	<0.001
*Vs. ALBI score*	0.591	0.354	Z = 6.943	<0.001
External validation set (2-year survival rate)				
Baseline model	0.846	/	/	/
*Vs. BCLC staging*	0.674	0.170	Z=2.537	0.001
*Vs. Child-Pugh class*	0.537	0.307	Z=5.030	<0.001
*Vs. ALBI score*	0.599	0.245	Z=3.997	<0.001
Integrated model	0.939	/	/	/
*Vs. Baseline model*	0.846	0.092	Z = 1.799	0.072
*Vs. BCLC staging*	0.674	0.263	Z = 4.451	<0.001
*Vs. Child-Pugh class*	0.537	0.400	Z = 7.638	<0.001
*Vs. ALBI score*	0.599	0.338	Z = 6.420	<0.001

AUC, area under curve; BCLC, Barcelona Clinic Liver Cancer; ALBI, Albumin-bilirubin ratio.

### 3.5 Sensitivity analysis and subgroup analysis

The time−dependent Cox model yielded results that were nearly identical to those of the original integrated model: HR for ORR = 0.49 (95% CI 0.28–0.85, P = 0.011), HR for AFP response=0.44 (95% CI 0.28–0.69, P<0.001). Hazard ratios for baseline variables were also unchanged (**Supplementary Table 8**). The C−index of the time-dependent model was 0.855 in both the training and external validation sets and 0.848 in the bootstrap validation, essentially unchanged from the original integrated model (**Supplementary Figure 6**). Patients who received conversion surgery or/and second-line therapy were censored at treatment initiation, and the model’s C-index was recalculated. The results showed that the changes were minimal (<0.01) and not statistically significant (all P > 0.05) (**Supplementary Table 9**), indicating that the main conclusions were robust to these interventions.

Subgroup analysis forest plots of each model in the training and external validation sets are detailed in Supplement **Supplementary Figures 7** and **S8**. For the baseline model, the C-indices ranged from 0.733 to 0.856 in the training set and 0.754 to 0.880 in the external validation set, remained stable overall. For the integrated model, the C-indices ranged from 0.818 to 0.906 in the training set and 0.792 to 0.917 in the external validation set, remained stable overall. These results demonstrate the model’s generalization ability across patients with different clinical features. Due to the small sample size and positive events in some subgroups, the results should be interpreted with caution.

### 3.6 Clinical benefits and risk stratification

**Figure 5:**
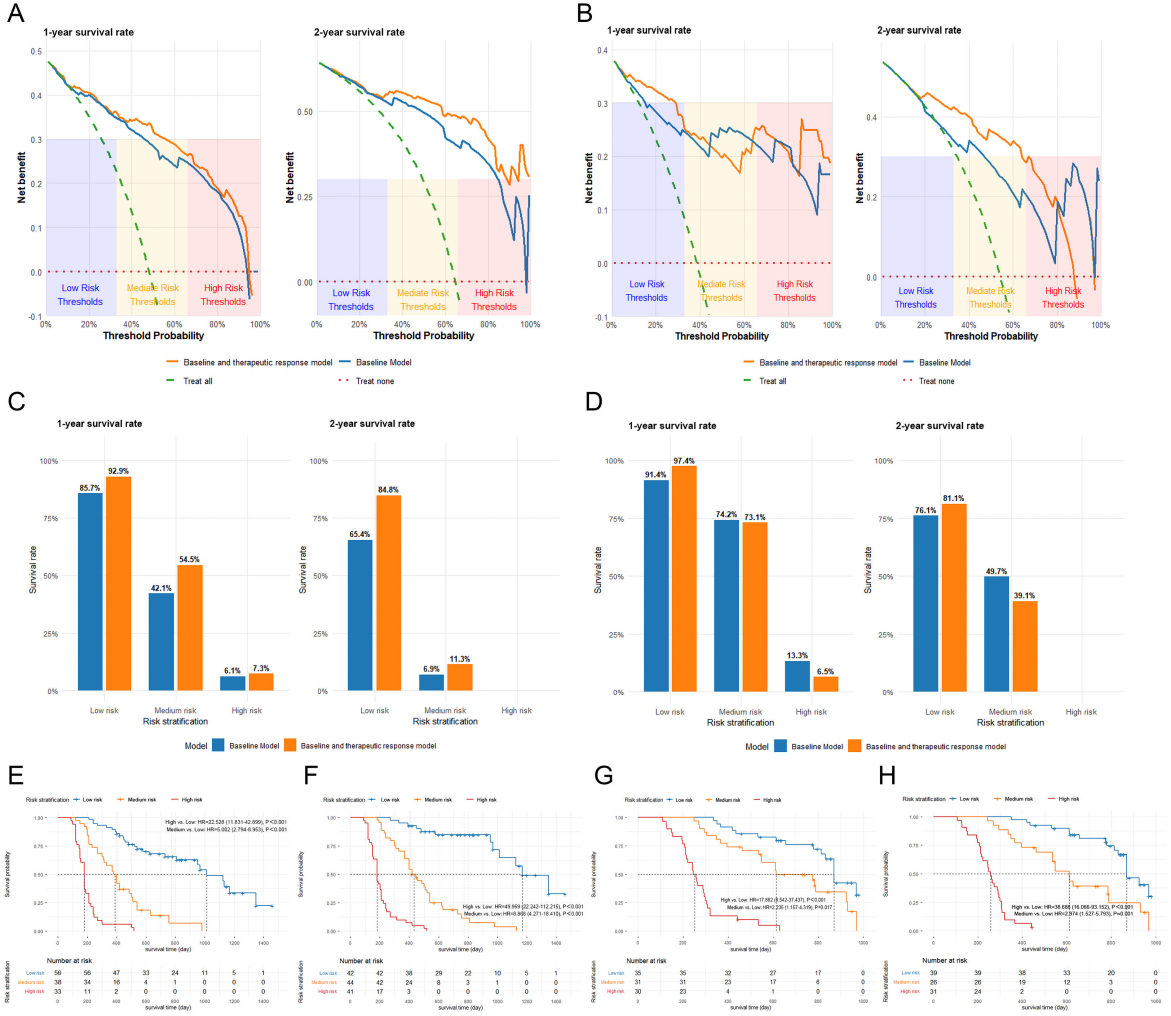
Clinical benefits and risk stratification. **(A)** Decision curve analysis of 1-year and 2-year survival rates in the training cohort; **(B)** Decision curve analysis of 1-year and 2-year survival rates in the external validation cohort; **(C)** The 1-year and 2-year survival rates histogram for risk stratification in the training cohort; **(D)** The 1-year and 2-year survival rates histogram for risk stratification in the external validation cohort; **(E)** Survival curves for risk groups based on risk stratification of the baseline model in the training cohort; **(F)** Survival curves for risk groups based on risk stratification of the integrated model in the training cohort; **(G)** Survival curves for risk groups based on risk stratification of the baseline model in the external validation cohort; **(H)** Survival curves for risk groups based on risk stratification of the integrated model in the external validation cohort. HR, hazard ratio.

Decision curve analysis showed the dynamic nomogram had a higher net benefit across thresholds than ‘Treat All’ and ‘Treat None’ in both training and validation cohorts (**Figures 5A, B**). Risk stratification into low- (0–33.3%), medium- (33.4–66.6%), and high-risk (66.7–100%) groups revealed progressively worse survival outcomes in both sets (baseline model: low-risk threshold=1.85, high-risk threshold=8.42; integrated model: low-risk threshold=2.03, high-risk threshold=12.61). The survival rate histograms for risk stratification in the training set and external validation set are shown in **Figures 5C, D**, respectively. The survival curves for risk stratification in the training set and external validation set are shown in **Figure 5E** (baseline model), **Figure 5F** (integrated model), and **Figure 5G** (baseline model), **Figure 5H** (integrated model), respectively.

### 3.7 Model interpretability and online deployment

**Figure 6:**
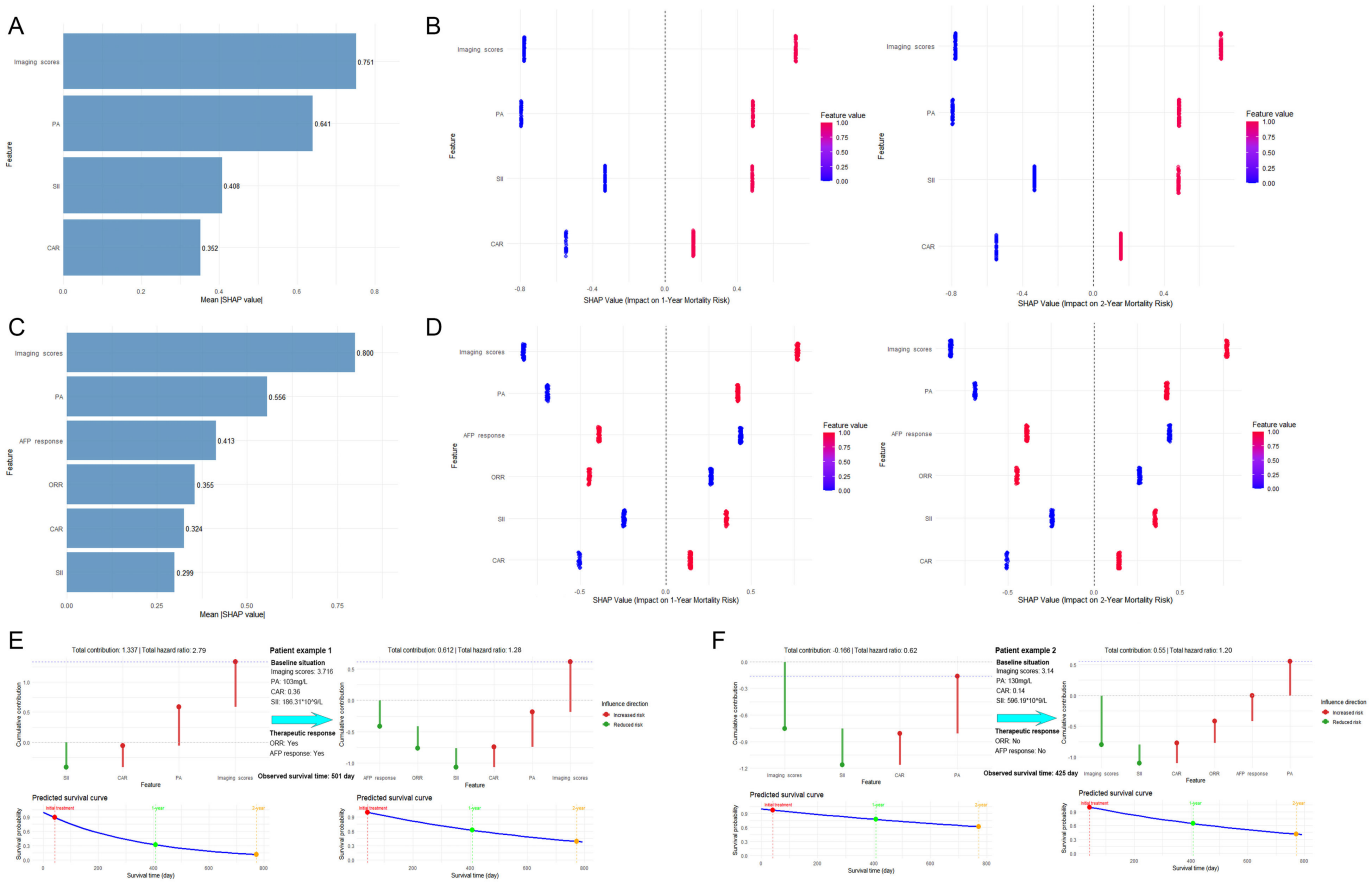
Global interpretation and personalized interpretation of the model. **(A)** The mean SHapley Additive exPlanations (SHAP) values of global feature importance in the baseline model; **(B)** The SHAP summary plots of 1-year and 2-year mortality risks in the baseline model; **(C)** The mean SHAP values of global feature importance in the integrated model; **(D)** The SHAP summary plots of 1-year and 2-year mortality risks in the integrated model; **(E)** Waterfall diagram of personalized risk interpretation for one patient from the training cohort; **(F)** Waterfall diagram of personalized risk interpretation for one patient from the external validation cohort. PA, prealbumin; SII, systemic Immune Inflammation index; CAR, C-reactive protein to albumin ratio; ORR, objective response rate; AFP, alpha-fetoprotein.

SHAP analysis identified the imaging score as the most influential predictor in both the baseline (**Figures 6A, B**) and integrated models (**Figures 6C, D**). The summary plot showed that all baseline characteristics were independent risk factors on 1-year and 2-year mortality risks; higher eigenvalues corresponded to more positive SHAP values and higher death risk contribution, while AFP response and ORR were independent protective factors.

**Figure 6E, F** shows two examples of localized, personalized interpretations for patients, respectively. The dynamic nomogram model has been deployed online as an R/Shiny application (the baseline model: https://tanhaoyang.shinyapps.io/liver-cancer-baseline/ and the integrated model: https://tanhaoyang.shinyapps.io/liver-cancer-joint/). The related analysis code is provided in **Supplementary File S1**. Screen captures of the online interface are shown in **Supplementary Figure 9**.

## 4 Discussion

In this study, we developed and validated a dynamic nomogram combining a Gd-EOB-DTPA-enhanced MRI semantic score with clinical features to provide a noninvasive prognosis for uHCC patients undergoing triple therapy. The baseline model predicts survival risk before treatment, while the integrated model updates this risk after initial assessment. This dynamic approach effectively identifies medium- and high-risk patients with poor prognoses which may help inform clinical decisions. The dynamic nomogram numerically improved staging systems such as BCLC and liver function grades. The imaging score, based on five MRI semantic features, showed excellent discrimination, with high-risk patients (>3.52) having worse outcomes. The score was the strongest mortality predictor. Recent studies have confirmed that these specific imaging features are associated with high-risk pathological characteristics indicative of poor prognosis (20). This association with aggressive tumor biology may explain how these imaging features predict prognosis in uHCC patients on triple therapy. In the external validation set, the baseline model’s calibration for 2-year survival was poor (calibration slope 0.379, intercept 0.375, P = 0.035), indicating a systematic discrepancy between model-predicted and observed survival probabilities. Significant differences in high-risk nutritional status and high-risk inflammatory levels between the training and external validation sets may explain the poor external calibration of the baseline model. The training set had more patients with high-risk nutrition and high-risk inflammation. The model may have relied heavily on these features to predict the risk of death during training. When applied to the external validation set, the predictions exhibited systematic bias due to differences in the distribution of these features. Although PA and CAR distributions differed, no significant differences were observed in other key variables. This suggests that the model’s predictions, based on tumor imaging characteristics and early treatment response, were consistent across both groups. The integrated model remained reliable, with an external validation C-index of 0.855, demonstrating its robustness to such variations. In contrast, the integrated model maintained good 2-year calibration in external validation (slope 0.991, p=0.095), suggesting that incorporating early treatment response can effectively improve the accuracy of long-term prediction. In addition, the short follow-up time of the validation set means that more patients remain in the deletion state at 2 years (no deaths are observed). This increases the likelihood that the calibration curve will deviate from the ideal line. Further validation is needed in a larger, more detailed, and longer follow-up prospective cohort.

While Xu et al. demonstrated excellent predictive accuracy with their multimodal fusion system and longitudinal CT-based radiomics model in large multicenter cohorts (17, 18), our study offers unique clinical value as the first dynamic prognostic model that combines Gd-EOB-DTPA-enhanced MRI semantic features with early treatment response. Our model, first, uses semantic features that radiologists routinely interpret, requiring no extra radiomic extraction or deep learning, which allows for swift clinical decisions. Second, by incorporating early treatment response indicators (ORR and AFP response), the model boosted the C-index from 0.822 to 0.855, with significant improvements in reclassification metrics (NRI and IDI, P < 0.05). This aligns with radiomics strategies that improve prognosis by combining radiomic scores with mRECIST, but our method is simpler, relying only on dichotomous semantic features. Both approaches complement each other: detailed radiomics can be used when resources allow, while our semantic model provides a quick screening tool in routine settings.

Previous studies have established that inflammatory biomarkers can predict non-response and PFS in HCC patients receiving HAIC (35). Our study links systemic inflammatory indices, like high SII and CAR, with tumor prognosis. These are independent risk factors for OS and key in our predictive model, highlighting inflammation’s role in outcomes. Elevated inflammation before triple therapy may lead to shorter survival. Using SII and CAR, obtained from routine tests, could help in prognosis and personalized treatment decisions.

PA, like ALB, indicates nutritional status, but with a shorter half-life, it reflects changes more quickly. Studies show PA’s prognostic value in HCC patients (36, 37). Our results confirm this, identifying <150 mg/L as a key cut-off for predicting survival. Low PA was linked to poor prognosis and was vital in our model. The exact mechanism linking nutritional status and prognosis requires further study.

The correlation between early tumor response and long-term prognosis is also noteworthy. Elevated baseline AFP levels are widely recognized as an independent risk factor for poor prognosis in patients with HCC (38). Our study found no significant association between pre-treatment AFP levels and OS, likely due to tumor heterogeneity. However, a decrease in AFP after treatment was strongly associated with better prognosis. Why does the AFP response outperform baseline AFP? Baseline AFP levels are heterogeneous and do not reliably distinguish aggressive from indolent tumors; many aggressive HCCs are AFP−negative. In contrast, a significant decline in AFP after two cycles directly reflects tumor sensitivity to the triple regimen, providing a real−time functional assessment of treatment efficacy. This biological rationale explains why dynamic response indicators are superior to static baseline values for predicting long−term prognosis. Our research also confirmed that the integrated model, including post-treatment response indicators, significantly enhances baseline model predictions (C-index, NRI, and IDI). These findings underscore the clinical importance of monitoring early tumor response but require validation in prospective trials.

Several limitations of this study should be acknowledged. First, this retrospective multicenter analysis included two Chinese centers with a modest external validation set, which may limit generalizability. Despite good retrospective performance, the model’s clinical utility needs to be confirmed through prospective studies to determine whether it improves treatment decisions and patient outcomes. Second, the model relies on Gd-EOB-DTPA-enhanced MRI, which may not be available everywhere, limiting its universal use. Future studies should assess whether similar features can be extracted using non-specific gadolinium agents or other imaging methods, such as CT, to broaden its applicability. Additionally, our model, developed for triple therapy, predicts overall survival rather than immunotherapy efficacy alone. It lacks immune−related biomarkers such as PD−L1, TMB, or immune microenvironment features, so it can’t determine the benefit of immune checkpoint inhibitors. Future studies should assess how these biomarkers contribute to the effects of individual treatment components. Then, our inclusion criteria required completion of at least two cycles of therapy, which may introduce selection bias by excluding patients who discontinued early because of disease progression, severe adverse events, or death. Therefore, the model’s conclusions cannot be generalized directly to all uHCC patients receiving triple therapy, particularly those who are unsuitable for or unable to complete the induction phase. Future prospective studies are needed to validate the model’s performance in broader populations, including those with early treatment failure. Additionally, the optimal thresholds for continuous variables were determined based on the data, with sensitivity analyses indicating robustness; external validation is recommended. Subsequent treatments, such as second-line therapy or conversion surgery, may influence long-term survival, although sensitivity analyses with censoring showed little effect. Finally, the study primarily includes HBV-related HCC patients with a uniform cause. Given regional differences in HCC etiologies, such as HCV, alcohol, and fatty liver, the model’s effectiveness in other populations needs to be tested. Future validation in diverse international cohorts is necessary.

In conclusion, we developed and validated a dynamic nomogram that integrates baseline features, including an imaging score derived from Gd-EOB-DTPA-enhanced MRI semantic features, and therapeutic response to predict long-term prognosis in uHCC patients undergoing triple therapy, thereby providing a noninvasive tool for risk stratification and prognostic assessment in uHCC patients receiving triple therapy, with the potential to inform personalized treatment decisions after further prospective validation.

## Data Availability

The original contributions presented in the study are included in the article/**Supplementary Material**. Further inquiries can be directed to the corresponding authors.
