# Supplementary material for: Dynamic nomogram integrating Gd-EOB-DTPA enhanced MRI semantic features, nutritional-inflammatory indices, and early treatment response to predict long-term survival in unresectable HCC treated with interventional, targeted, and immunotherapy: a multicenter retrospective study
Source: Front Immunol. 2026 Jul 1;17:1848104. doi: 10.3389/fimmu.2026.1848104 (PMC13368791; doi:10.3389/fimmu.2026.1848104)
Supplement: Supplementary file 1 [file Table1.docx]

**Appendix S1: MR scanning protocol and contrast agent dosage**

At Center 1 (train set), MRI scans were conducted using a 3.0-T system (MAGNETOM Prisma, Siemens Healthineers). At Center 2 (external validation set), a 3.0-T MRI was performed with a uMR780 system (Shanghai United Imaging Healthcare).

The MRI protocol consisted of T2-weighted 2d fast spin echo imaging, diffusion-weighted imaging with b values of 0, 50, 500, 800, 1000, and 1200 s/mm² using Siemens MAGNETOM Prisma, and 0, 200, 800, and 1000 s/mm² with United Imaging uMR780, along with ADC maps. It also included T1-weighted dual gradient-echo in- and opposed-phase imaging and dynamic T1-weighted 3d gradient-echo imaging, performed before and after gadolinium-based agent administration (Primovist®, Bayer Pharma AG). These scans covered late arterial, portal venous (60 s), transitional (3 min), and hepatobiliary (20 min) phases. Arterial phase images were acquired either with trigger 7 seconds after contrast reaches the celiac trunk or via a multiple arterial phase (MAP) technique. MAP images were obtained with an 18-second breath hold 20 seconds post-contrast, then reconstructed at 3-second temporal resolution. The contrast agent was infused at 1 ml/sec for a total of 0.025 mmol/kg, followed by a 20 ml saline flush. Details of the MRI sequences are listed in Table S1.

| **Table S1.** **MRI sequences and parameters** | | | | |
| --- | --- | --- | --- | --- |
| **Sequence** | **T1-weighted IP and OP imaging** | **Dynamic T1-weighted 3D GRE** | **T2-weighted 2D FSE** | **Diffusion-weighted imaging^†^** |
| **Siemens MAGNETOM Prisma (18-channel body array coil)-institution 1 (train set)** | | | | |
| Repetition time (ms) | 81 | 3.37 | 2200 | 5600 |
| Echo time (ms) | 2.72/1.4 | 1.33 | 77 | 68 |
| Flip angle (°) | 70 | 9 | 160 | 90 |
| Section thickness (mm) | 6 | 3.0 | 6 | 6 |
| Spacing (mm) | 1.8 | - | 1.8 | 1.8 |
| Matrix size | 352×286 | 352×246 | 320×224 | 100×76 |
| Field of view (mm^2^) | 400×325 | 380×313.12 | 380×380 | 380×289 |
| Acquisition time (s) | 24 | 20 | 80 | 233 |
| Fat suppression | No | Yes | Yes | Yes |
| Phase enc. Dir. | A>>P | A>>P | R>>L | A>>P |
| Voxel size (mm^3^) | 1.1×1.1×3.0 | 1.1×1.1×3.0 | 1.2×1.2×6.0 | NA |
| **uMR780 (48-channel body array coil) -institution 2 (External validation set)** | | | | |
| Repetition time (ms) | 150 | 3.07 | 2200 | 5000 |
| Echo time (ms) | 2.5/1.3 | 1.41 | 77 | Minimum |
| Flip angle (°) | 70 | 10 | - | 90 |
| Section thickness (mm) | 6 | 5 | 6 | 7 |
| Spacing (mm) | 2 | - | 1.8 | 2 |
| Matrix size | 288×192 | 320×240 | 320×320 | 160×128 |
| Field of view (mm^2^) | 420×420 | 420×315 | 380×380 | 380×342 |
| Acquisition time (s) | 31 | 17 | 36188 | RG |
| Fat suppression | No | Yes | Yes | Yes |
| Phase enc. Dir. | A>>P | A>>P | R>>L | A>>P |
| Voxel size (mm^3^) | 1.7×1.3×5.0 | 1.7×1.3×5.0 | 1.2×1.2×6.0 | NA |
| MRI, magnetic resonance imaging; IP, in-phase; OP, opposed-phase; 3D, three-dimensional; GRE, gradient recall echo; 2D, two-dimensional; FSE, fast spin-echo; RG, respiratory gating.  †Images were acquired under free breath. | | | | |

| **Table S2. Definitions of imaging features.** | |
| --- | --- |
| **Imaging features** | **Definitions** |
| Tumor size | The maximum diameter on transverse images. |
| Tumor number | Number of unequivocal HCC. |
| Vascular invasion | Tumor thrombus in branches of portal vein or hepatic vein. (1) |
| Incomplete capsule | An absence or disruption of “capsule” in any imaging plane. (2) |
| Intratumoral necrosis | A nonenhancing region within a solid mass that cannot be explained by a cystic part, previous treatment, or bleeding inside the lesion. (3,4) |
| Irregular morphology | The tumor margin appears irregular and/or exhibits bulging, nodular projections, or infiltration into nearby tissues in any imaging plane. (5,6) |
| Intratumoral arteries | Discrete arteries visible within the tumor on arterial phase images. (5,7) |
| Rim enhancement | A subtype of arterial phase hyperenhancement characterized by the most pronounced enhancement at the tumor’s periphery during the arterial phase. (3,4) |
| Peritumoral low signal | Presence of an irregular, wedge-shaped, or flame-like hypointense area in the liver parenchyma outside the tumor margin during the hepatobiliary phase. (8,9) |
| Peritumoral enhancement | The peritumoral enhancement in the arterial phase was a band-like enhancement surrounding the tumor during the arterial phase. (1) |
| Satellite lesions | Presence of separate unequivocal HCC(s) within 2 cm of the border of the dominant tumor. (10) |
| Mosaic sign | The mosaic sign appeared as random internal nodular signals or compartmental features within the tumor and was more clearly seen on T2-weighted images. (11,12) |
| For multi-focal HCC, the feature was considered present if any lesion met the criteria. | |

| **Table S3. Baseline characteristics of all patients included in the study.** | | | | | |
| --- | --- | --- | --- | --- | --- |
| Characteristic | Total cohort  (n=223) | Training cohort  (n=127) | Validation cohort  (n=96) | Statistic | *P value |
| Gender, n(%) |  |  |  | χ²=0.108 | 0.742 |
| *Female* | 19(8.5%) | 12(9.4%) | 7(7.3%) |  |  |
| *Male* | 204(91.5%) | 115(90.6%) | 89(92.7%) |  |  |
| Age (y), Mean±SD | 56.1±11.4 | 56.4±10.9 | 55.7±12.2 | t=0.450 | 0.653 |
| BMI, Median(IQR) | 23.1(21.6-24.9) | 23.4(21.2-25.6) | 22.8(21.8-23.8) | Z=-1.516 | 0.130 |
| Viral hepatitis, n(%) |  |  |  |  |  |
| *Hepatitis B virus* | 178(79.8%) | 101(79.5%) | 77(80.2%) | χ²=0.001 | 0.999 |
| *Hepatitis C virus* | 45(20.2%) | 26(20.5%) | 19(19.8%) |  |  |
| Cirrhosis, n(%) |  |  |  | χ²=0.124 | 0.725 |
| *Absence* | 69(30.9%) | 41(32.3%) | 28(29.2%) |  |  |
| *Presence* | 154(69.1%) | 86(67.7%) | 68(70.8%) |  |  |
| CNLC, n(%) |  |  |  | χ²=1.022 | 0.312 |
| *IIb* | 72(32.3%) | 45(35.4%) | 27(28.1%) |  |  |
| *IIIa* | 151(67.7%) | 82(64.6%) | 69(71.9%) |  |  |
| BCLC, n(%) |  |  |  | χ²=1.022 | 0.312 |
| *B* | 72(32.3%) | 45(35.4%) | 27(28.1%) |  |  |
| *C* | 151(67.7%) | 82(64.6%) | 69(71.9%) |  |  |
| PS score, n(%) |  |  |  | χ²=0.054 | 0.816 |
| *0* | 177(79.4%) | 102(80.3%) | 75(78.1%) |  |  |
| *1* | 46(20.6%) | 25(19.7%) | 21(21.9%) |  |  |
| Child-Pugh score, n(%) |  |  |  | χ²=0.284 | 0.594 |
| *A(5-6)* | 188(84.3%) | 109(85.8%) | 79(82.3%) |  |  |
| *B(7-8)* | 35(15.7%) | 18(14.2%) | 17(17.7%) |  |  |
| ALBI, n(%) |  |  |  | χ²=0.031 | 0.861 |
| *1(≤-2.60)* | 172(77.1%) | 99(78.0%) | 73(76.0%) |  |  |
| *2(-2.60~-1.39)* | 51(22.9%) | 28(22.0%) | 23(24.0%) |  |  |
| ALT(U/L), Median(IQR) | 43.0(27.5-67.0) | 43.0(27.0-68.5) | 43.0(30.0-65.2) | Z=-0.251 | 0.802 |
| AST(U/L), Median(IQR) | 59.0(39.0-93.5) | 65.0(41.0-97.5) | 57.0(38.0-91.2) | Z=-1.155 | 0.248 |
| ALP(U/L), Median(IQR) | 131.0(98.5-183.5) | 128.0(97.0-180.5) | 144.5(100.5-213.0) | Z=-1.000 | 0.317 |
| GGT(U/L), Median(IQR) | 171.0(89.0-307.5) | 165.0(89.0-300.0) | 173.5(91.0-319.2) | Z=-0.446 | 0.655 |
| TBil(umol/L), Median(IQR) | 15.7(12.2-22.4) | 15.8(12.5-22.8) | 15.7(11.2-21.9) | Z=-0.445 | 0.656 |
| ALB(g/L), Mean±SD | 39.9±4.7 | 40.3±4.7 | 39.3±4.6 | t=1.466 | 0.144 |
| PA(mg/L), Median(IQR) | 131.0(96.0-184.0) | 118.0(92.0-184.0) | 156.0(101.2-184.0) | Z=-0.998 | 0.318 |
| PT(s), Median(IQR) | 13.8(13.1-14.5) | 13.8(13.1-14.4) | 13.6(13.1-14.7) | Z=-0.604 | 0.546 |
| **Inflammation index** | | | | | |
| CRP(mg/L), Median(IQR) | 10.9(6.7-35.1) | 11.2(5.1-34.6) | 10.8(7.3-35.3) | Z=-1.515 | 0.130 |
| SII(*10^9/L), Median(IQR) | 575.1(308.7-1003.7) | 559.1(308.7-1035.1) | 592.4(336.7-951.7) | Z=-0.313 | 0.754 |
| NLR, Median(IQR) | 3.42(2.38-5.72) | 3.45(2.23-5.78) | 3.24(2.50-5.17) | Z=-0.423 | 0.672 |
| PLR, Median(IQR) | 154.0(101.2-224.6) | 151.7(100.0-214.5) | 155.3(106.7-265.6) | Z=-1.008 | 0.313 |
| CAR, Median(IQR) | 0.25(0.07-0.79) | 0.31(0.12-0.79) | 0.20(0.07-0.75) | Z=-1.619 | 0.105 |
| PNI, Mean±SD | 45.7±5.7 | 46.1±5.6 | 45.3±5.8 | t=1.031 | 0.304 |
| **Tumor characteristics** | | | | | |
| Maximum diameter (cm), Median(IQR) | 8.1(5.5-10.8) | 7.5(5.5-10.5) | 8.4(5.7-11.3) | Z=-0.935 | 0.350 |
| Number, n(%) |  |  |  | χ²=0.286 | 0.593 |
| *Single* | 53(23.8%) | 28(22.0%) | 25(26.0%) |  |  |
| *Multiple* | 170(76.2%) | 99(78.0%) | 71(74.0%) |  |  |
| Six-and-Twelve, n(%) |  |  |  | χ²=0.937 | 0.626 |
| *G1(≤6)* | 25(11.2%) | 16(12.6%) | 9(9.4%) |  |  |
| *G2(6-12)* | 123(55.2%) | 71(55.9%) | 52(54.2%) |  |  |
| *G3(>12)* | 75(33.6%) | 40(31.5%) | 35(36.5%) |  |  |
| AFP positive, n(%) | 193(86.5%) | 109(85.8%) | 84(87.5%) | χ²=0.027 | 0.869 |
| AFP level(ng/mL), n(%) |  |  |  | χ²=1.780 | 0.411 |
| *<400* | 92(41.3%) | 57(44.9%) | 35(36.5%) |  |  |
| *400-1210* | 64(28.7%) | 33(26.0%) | 31(32.3%) |  |  |
| *>1210* | 67(30.0%) | 37(29.1%) | 30(31.2%) |  |  |
| **Early treatment response (after two cycles of treatment)** | | | | | |
| mRECIST criterion, n(%) |  |  |  | χ²=4.792 | 0.188 |
| *Complete response* | 16(7.2%) | 11(8.7%) | 5(5.2%) |  |  |
| *Partial response* | 73(32.7%) | 37(29.1%) | 36(37.5%) |  |  |
| *Stable disease* | 123(55.2%) | 75(59.1%) | 48(50.0%) |  |  |
| *Progression disease* | 11(4.9%) | 4(3.1%) | 7(7.3%) |  |  |
| Objective response rate, n(%) | 88(39.5%) | 47(37.0%) | 41(42.7%) | χ²=0.524 | 0.469 |
| Disease control rate, n(%) | 211(94.6%) | 122(96.1%) | 89(92.7%) | χ²=0.639 | 0.424 |
| AFP response rate, n(%) | 114(51.1%) | 67(52.8%) | 47(49.0%) | χ²=0.182 | 0.670 |
| **Subsequent treatments** | | | | | |
| Second-line drug, n (%) | 51(22.9%) | 28(22.0%) | 23(23.9%) | χ²=0.117 | 0.732 |
| Conversion surgery, n(%) | 21(9.4%) | 15(11.8%) | 6(6.2%) | χ²=1.384 | 0.239 |
| **Follow up** | | | | | |
| Median time(month), IQR | 21.5(16.5-28.8) | 24.6(17.7-29.5) | 20.4(16.5-24.4) | Z=1.623 | 0.036 |
| ∇Distant metastasis, n(%) | 64(28.7%) | 35(27.6%) | 29(30.2%) | χ²=0.080 | 0.777 |
| ∇Tumor progression, n(%) | 201(90.1%) | 113(89.0%) | 88(91.7%) | χ²=0.194 | 0.660 |
| ∇Death, n(%) | 157(70.4%) | 91(71.7%) | 66(68.8%) | χ²=0.104 | 0.747 |
| BMI, Body Mass Index; CNLC, China Liver Cancer; BCLC, Barcelona Clinic Liver Cancer; ALT, Alanine asninotrasferase; AST, Aspartic transaminase; ALP, alkaline phosphatase; GGT, gamma-glutamyl transpeptidase; TBil, Total Bilirubin; ALB, albumin; PA, Prealbumin; PT, Prothrombin Time; ALBI, Albumin-bilirubin ratio; AFP, Alpha-fetoprotein; CRP, C-reactive protein; SII, Systemic Immune Inflammation index; NLR,Neutrophil to Lymphocyte Ratio; PLR, Platelet to Lymphocyte Ratio; CAR, CRP to Albumin Ratio; PNI, Prognosis Nutritional Index; SD, Standard deviations; IQR, Interquartile Range.  * Comparison between training set and validation set.  ∇ Follow up period | | | | | |

| **Table S4. Observer consistency evaluation of image features.** | | | | |
| --- | --- | --- | --- | --- |
| **Imaging features** | **Kappa value** | **SE** | **95%CI** | **P*** |
| Vascular invasion | 0.949 | 0.029 | 0.892-1.006 | <0.001 |
| Incomplete capsule | 0.921 | 0.035 | 0.852-0.990 | <0.001 |
| Intratumoral necrosis | 0.905 | 0.041 | 0.825-0.985 | <0.001 |
| Irregular morphology | 0.905 | 0.038 | 0.831-0.979 | <0.001 |
| Intratumoral arteries | 0.936 | 0.032 | 0.873-0.999 | <0.001 |
| Rim enhancement | 0.915 | 0.037 | 0.842-0.988 | <0.001 |
| Peritumoral low signal | 0.934 | 0.033 | 0.869-0.999 | <0.001 |
| Peritumoral enhancement | 0.918 | 0.036 | 0.847-0.989 | <0.001 |
| Satellite lesions | 0.930 | 0.040 | 0.852-1.008 | <0.001 |
| Mosaic sign | 0.914 | 0.038 | 0.840-0.988 | <0.001 |
| SE, Standard error; CI, Confidence interval.  * The Kappa value is significantly different from 0 (indicating that consistency is not generated randomly). | | | | |

| **Table S5. The optimal cut-off values of continuous variables.** | | | | |
| --- | --- | --- | --- | --- |
| **Variables** | **Cut-off value** | **Maximal statistic** | **Hazard ratio (95%CI)** | **P value** |
| ALT (U/L) | 49 | 1.815 | 0.745 (0.484-1.147) | 0.1809 |
| AST (U/L) | 54 | 2.275 | 1.678 (1.084-2.598) | 0.0204 |
| ALP (U/L) | 122 | 2.516 | 1.703 (1.121-2.585) | 0.0125 |
| GGT (U/L) | 165 | 3.029 | 1.914 (1.257-2.915) | 0.0025 |
| TBil (umol/L) | 12.1 | 3.157 | 2.278 (1.305-3.974) | 0.0038 |
| PT (s) | 13.1 | 2.929 | 2.080 (1.224-3.533) | 0.0068 |
| PA (mg/L) | 150 | 5.668 | 0.264 (0.160-0.435) | <0.0001 |
| PLR | 161.48 | 4.025 | 2.438 (1.608-3.696) | <0.0001 |
| NLR | 4.17 | 4.790 | 3.080 (2.014-4.709) | <0.0001 |
| CAR | 0.08 | 4.813 | 3.714 (1.965-7.021) | 0.0001 |
| SII (*10^9/L) | 746.44 | 5.311 | 3.806 (2.490-5.816) | <0.0001 |
| PNI | 48 | 2.406 | 0.581 (0.363-0.929) | 0.0234 |
| ALT, Alanine asninotrasferase; AST, Aspartic transaminase; ALP, alkaline phosphatase; GGT, gamma-glutamyl transpeptidase; TBil, Total Bilirubin; PA, Prealbumin; PT, Prothrombin Time; SII, Systemic Immune Inflammation index; NLR,Neutrophil to Lymphocyte Ratio; PLR, Platelet to Lymphocyte Ratio; CAR, C-reactive protein to Albumin Ratio; PNI, Prognosis Nutritional Index. | | | | |

| **Table S6. The results of the continuous NRI and IDI in the training set.** | | | | |
| --- | --- | --- | --- | --- |
|  | **Estimate** | **Lower** | **Upper** | **P value** |
| **1-year survival rate after two cycles treatment** | | | | |
| NRI | 0.567 | 0.272 | 0.996 | 0.002 |
| NRI(+) | 0.082 | 0.007 | 0.621 |  |
| NRI(-) | 0.485 | 0.083 | 0.657 |  |
| Pr(Up \| Case) | 0.541 | 0.504 | 0.810 |  |
| Pr(Down \| Case) | 0.459 | 0.190 | 0.496 |  |
| Pr(Up \| Control) | 0.742 | 0.542 | 0.828 |  |
| Pr(Down \| Control) | 0.258 | 0.172 | 0.458 |  |
| IDI | 0.050 | 0.001 | 0.100 | 0.040 |
| **2-year survival rate after two cycles treatment** | | | | |
| NRI | 0.653 | 0.392 | 1.417 | 0.012 |
| NRI(+) | 0.235 | 0.080 | 0.648 |  |
| NRI(-) | 0.418 | 0.132 | 0.928 |  |
| Pr(Up \| Case) | 0.623 | 0.531 | 0.826 |  |
| Pr(Down \| Case) | 0.388 | 0.175 | 0.459 |  |
| Pr(Up \| Control) | 0.698 | 0.566 | 0.978 |  |
| Pr(Down \| Control) | 0.279 | 0.047 | 0.434 |  |
| IDI | 0.100 | 0.015 | 0.180 | 0.032 |
| NRI, net reclassification index; IDI, integrated discrimination improvement. | | | | |

| **Table S7. The Hosmer-Lemeshow of calibration curves for survival rates.** | | | | | | |
| --- | --- | --- | --- | --- | --- | --- |
| **Survival probability** | **Slope** | **Intercept** | **MSE** | **Brier Score** | **Integrated Brier Score** | **P value*** |
| ***Training set*** | | | | | | |
| **Baseline model** |  |  |  |  | 0.108 |  |
| 1-year | 0.942 | 0.044 | 0.001 | 0.136 | / | 0.419 |
| 2-year | 1.007 | -0.007 | <0.001 | 0.104 | / | 0.145 |
| **Integrated model** |  |  |  |  | 0.087 |  |
| 1-year | 0.995 | 0.016 | 0.003 | 0.122 | / | 0.215 |
| 2-year | 1.005 | -0.004 | 0.002 | 0.080 | / | 0.158 |
| ***External validation set*** | | | | | | |
| **Baseline model** |  |  |  |  | 0.113 |  |
| 1-year | 1.067 | -0.055 | 0.005 | 0.106 | / | 0.174 |
| 2-year | 0.379 | 0.375 | 0.022 | 0.147 | / | 0.035 |
| **Integrated model** |  |  |  |  | 0.097 |  |
| 1-year | 0.852 | 0.130 | 0.003 | 0.092 | / | 0.169 |
| 2-year | 0.991 | -0.030 | 0.004 | 0.106 | / | 0.095 |
| ***Bootstrap set (Mean value)*** | | | | | | |
| **Baseline model** |  |  |  |  | 0.104 |  |
| 1-year | 0.939 | 0.045 | 0.001 | 0.133 | / | 0.259 |
| 2-year | 1.002 | -0.006 | <0.001 | 0.100 | / | 0.132 |
| **Integrated model** |  |  |  |  | 0.082 |  |
| 1-year | 0.950 | 0.036 | 0.001 | 0.117 | / | 0.174 |
| 2-year | 0.989 | -0.001 | <0.001 | 0.076 | / | 0.202 |
| MSE, mean standard error.  *Hosmer‑Lemeshow test P value. | | | | | | |

| **Table S8. Comparison of the original integrated Cox model and time‑dependent Cox model (sensitivity analysis for immortal time bias).** | | |
| --- | --- | --- |
| **Variables** | **Original Integrated Model**  **HR (95% CI)** | **Time‑Dependent Model**  **HR (95% CI)** |
| Imaging score (high vs. low) | 4.95 (2.86–8.57) | 4.95 (2.86–8.57) |
| PA (high vs. low) | 3.04 (1.79–5.16) | 3.04 (1.79–5.16) |
| SII (high vs. low) | 1.82 (1.13–2.94) | 1.82 (1.13–2.94) |
| CAR (high vs. low) | 1.91 (0.95–3.86) | 1.91 (0.95–3.86) |
| ORR (yes vs. no) | 0.49 (0.28–0.85) | 0.49 (0.28–0.85)* |
| AFP response (yes vs. no) | 0.44 (0.28–0.69) | 0.44 (0.28–0.69)* |
| C‑index (training) | 0.855 | 0.855 |
| C‑index (external validation) | 0.855 | 0.855 |
| *Time-dependent covariates.  PA, Prealbumin; SII, Systemic Immune Inflammation index; CAR, CRP to Albumin Ratio; AFP, Alpha-fetoprotein; ORR, Objective response rate; HR, Hazard ratio | | |

| **Table S9. Sensitivity analysis of model performance following censoring of patients who received conversion surgery or/and second-line therapy.** | | | | | |
| --- | --- | --- | --- | --- | --- |
| **Model** | **Dataset** | **C‑index (original)** | **C‑index (censored)** | **Δ (95%CI)** | **P value** |
| Baseline | Training | 0.822 | 0.820 | -0.002 (-0.055,0.057) | 0.999 |
| Baseline | External | 0.799 | 0.792 | -0.007 (-0.091,0.069) | 0.999 |
| Integrated | Training | 0.855 | 0.851 | -0.003 (-0.052, 0.047) | 0.999 |
| Integrated | External | 0.855 | 0.865 | 0.009 (-0.044, 0.059) | 0.999 |

| 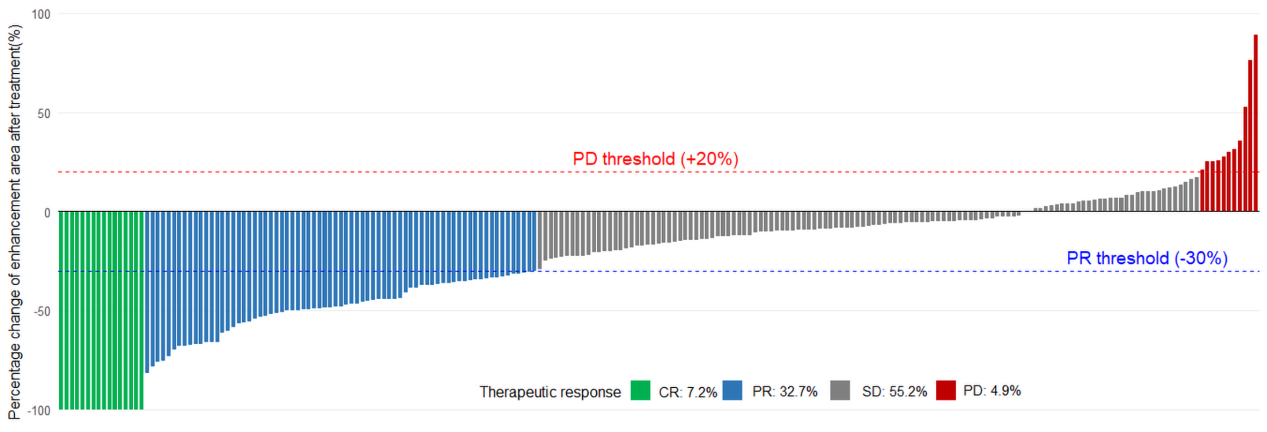 |
| --- |
| **Figure S1. Waterfall plot for the best percentage change in tumor enhancement area after two routine treatment cycles based on the mRECIST.** |

| 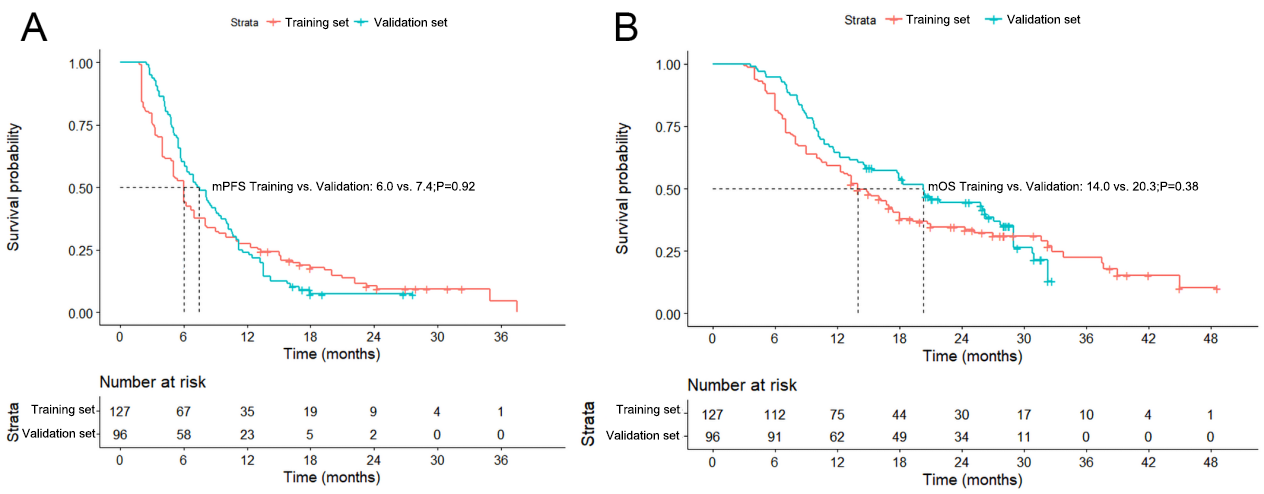 |
| --- |
| **Figure S2. Kaplan-Meier curves of A) progression free survival and B) overall survival between the training and validation sets.** |

| 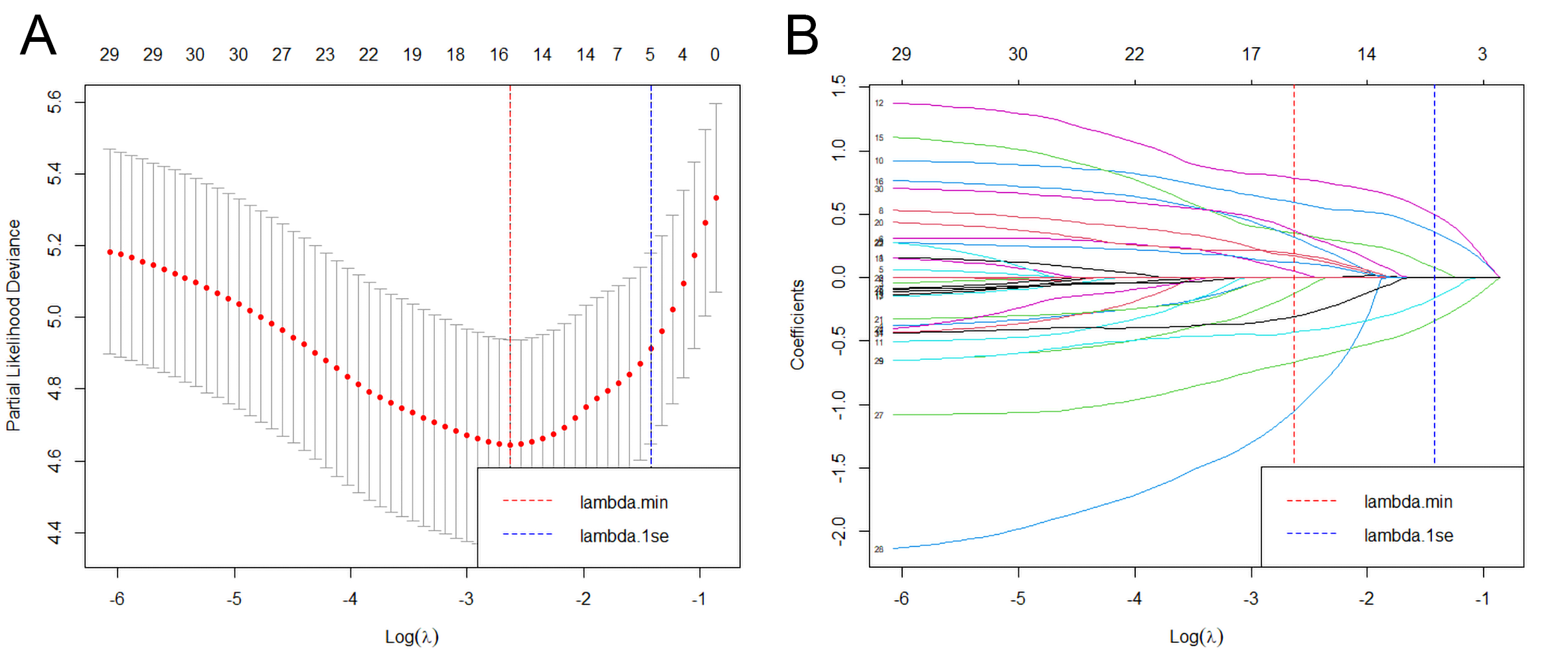 |
| --- |
| **Figure S3. Presentation of the results of the LASSO regression analysis. A) LASSO Regression Model Factor Selection; B) LASSO regression model screening variable trajectories.** |

| 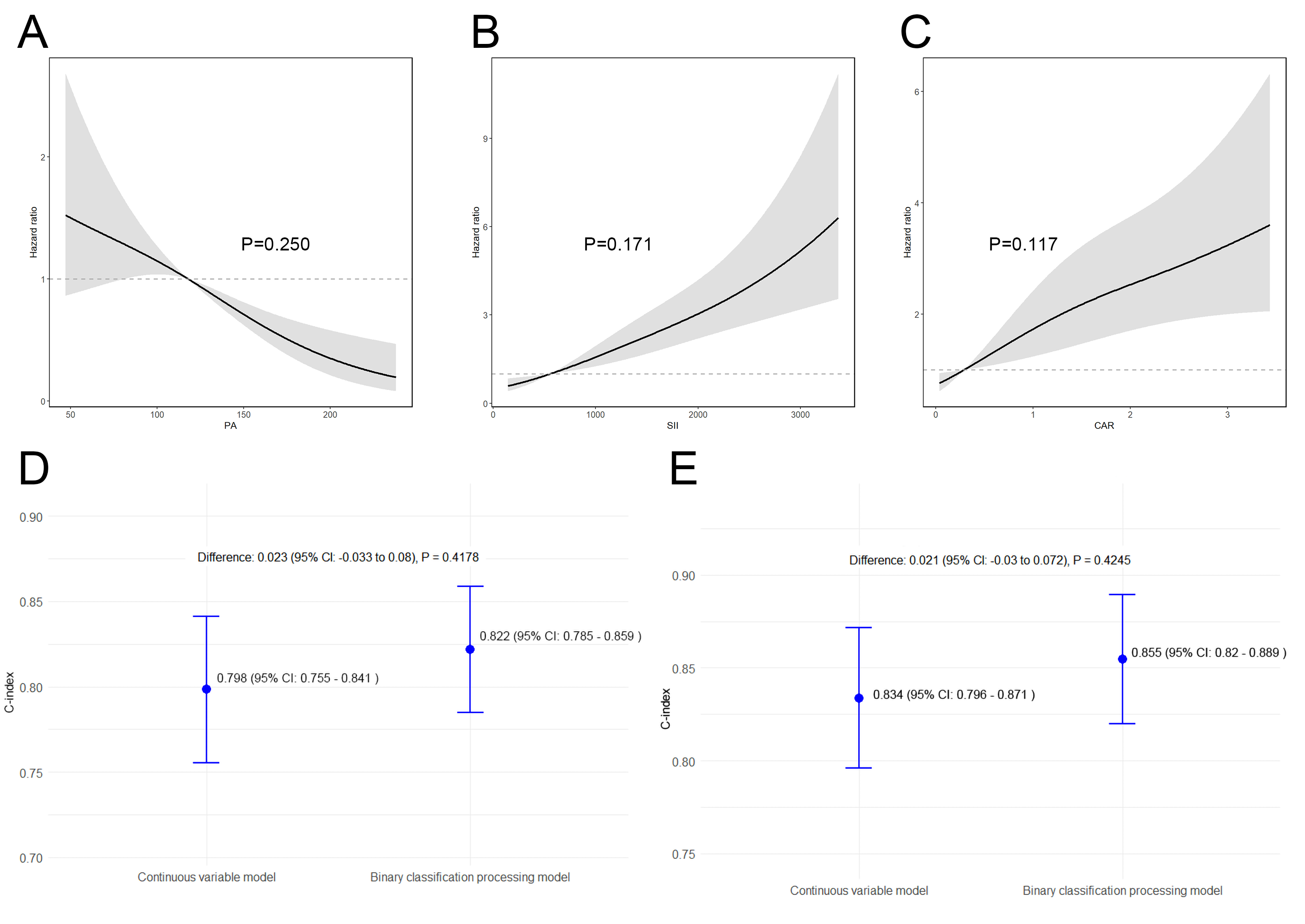 |
| --- |
| **Figure S4. Sensitivity analysis of continuous variables in the model. A-C) restrictive cubic splines of PA, SII, and CAR; D) baseline model; E) integrated model. PA, prealbumin; SII, systemic Immune Inflammation index; CAR, C-reactive protein to albumin ratio** |

| 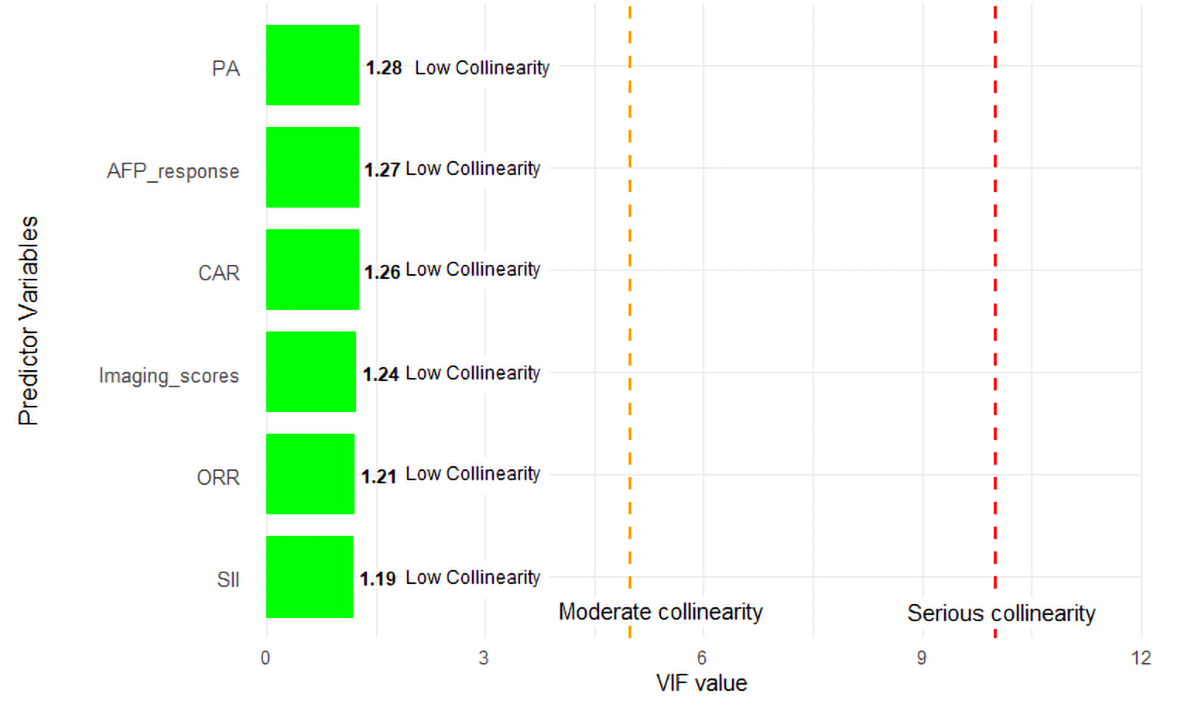 |
| --- |
| **Figure S5. Multicollinearity analysis of the nomogram model factors. VIF, variance inflation factor; PA, prealbumin; SII, systemic Immune Inflammation index; CAR, C-reactive protein to albumin ratio; ORR, objective response rate; AFP, alpha-fetoprotein** |

| 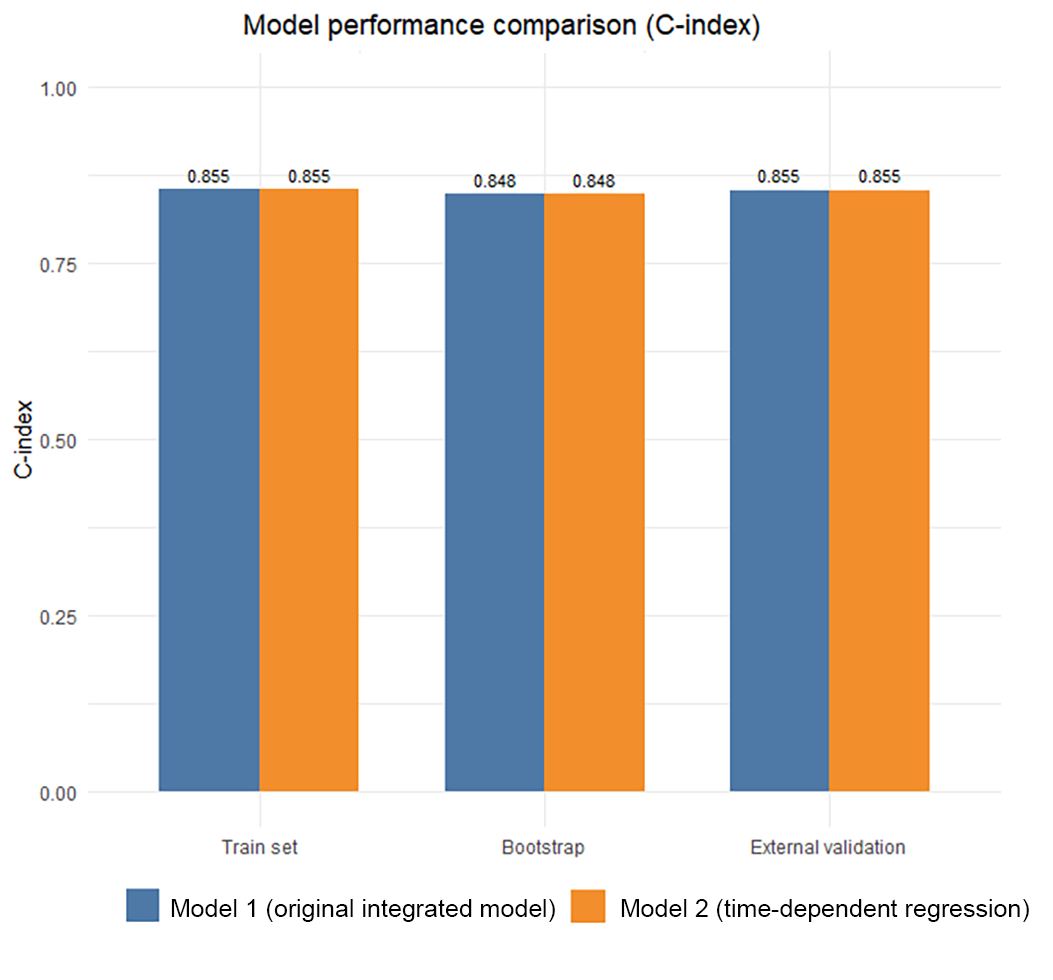 |
| --- |
| **Figure S6. Comparison of the C-index between the time-dependent model and original integrated model.** |

| 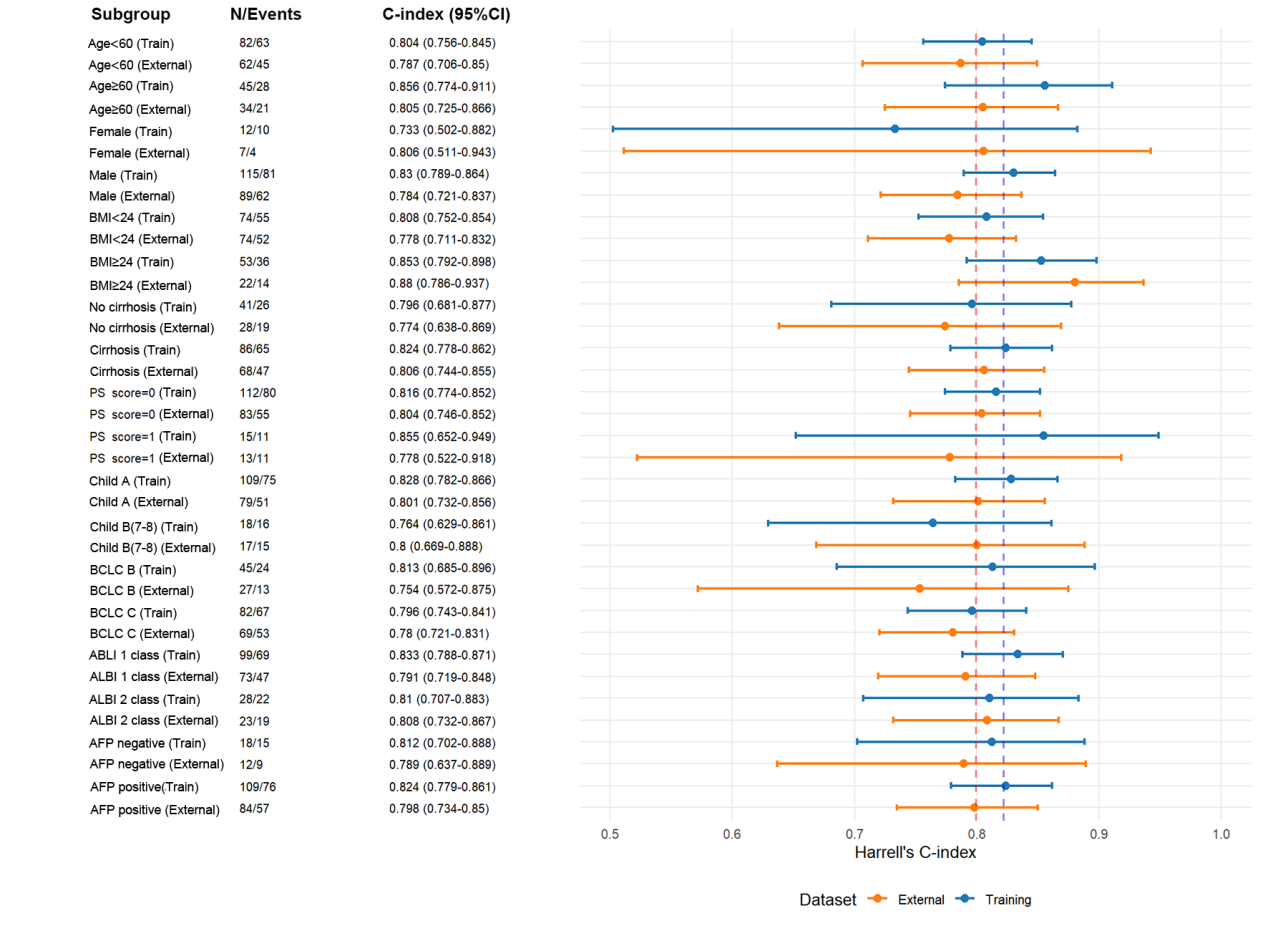 |
| --- |
| **Figure S7. Subgroup analysis of the baseline model in training and external validation sets.** |

| 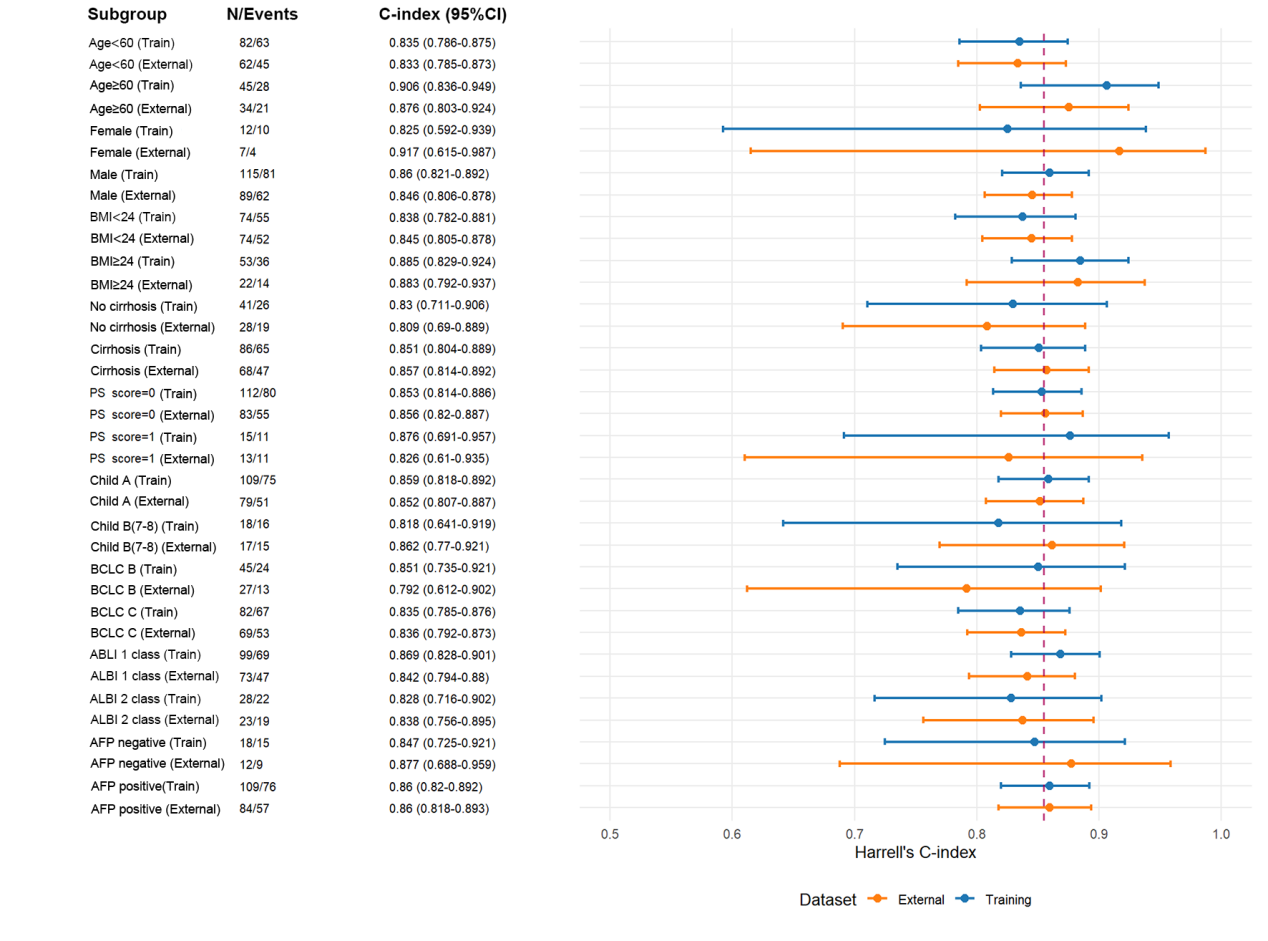 |
| --- |
| **Figure S8. Subgroup analysis of the integrated model in training and external validation sets.** |

| 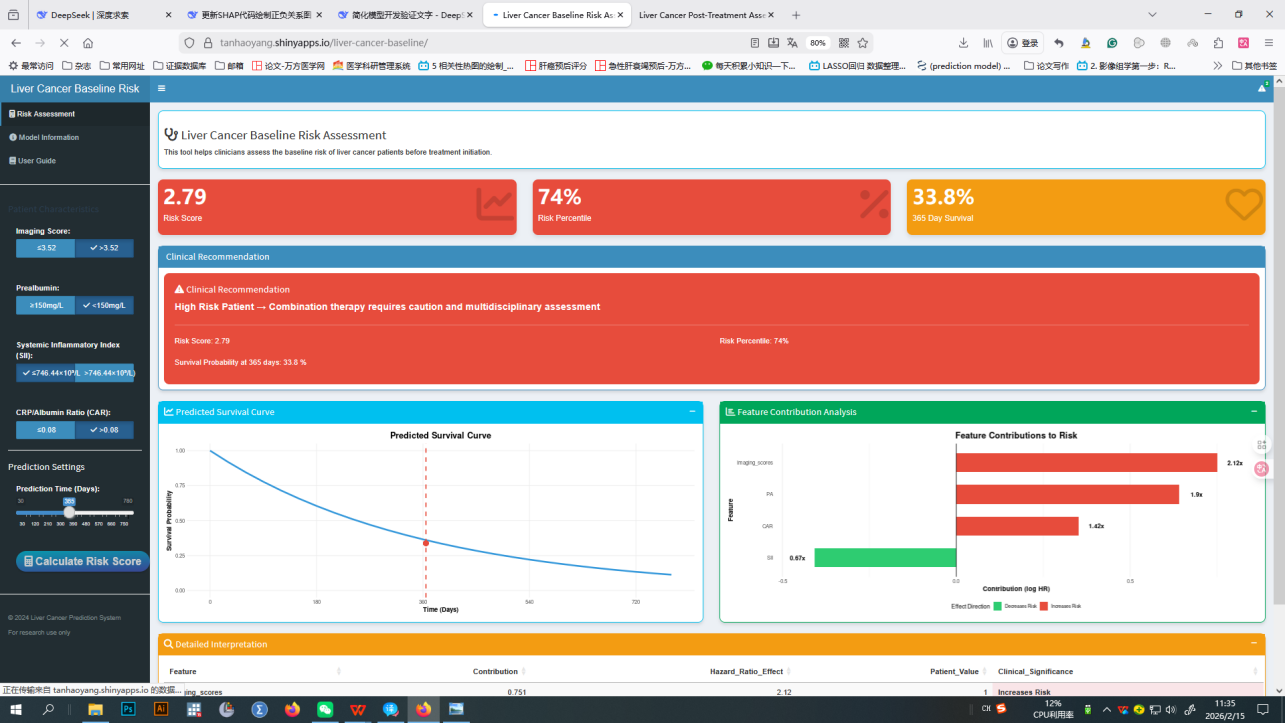  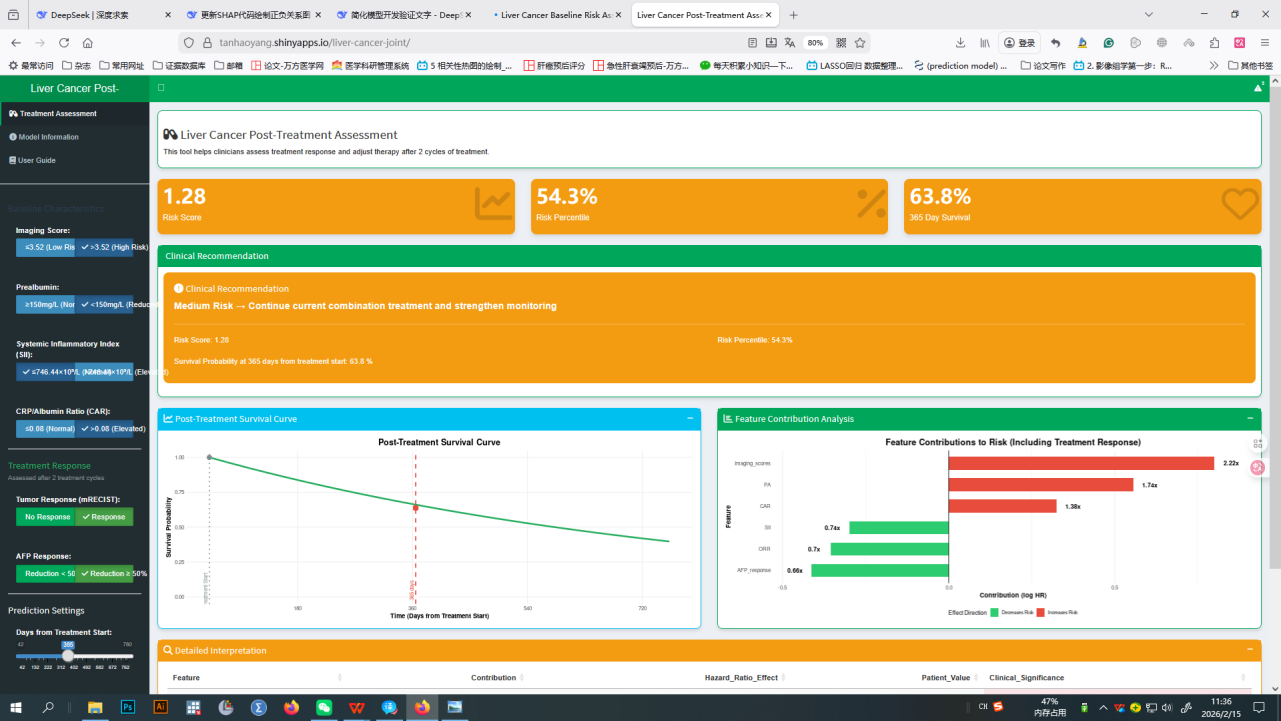 |
| --- |
| **Figure S9. Screen capture of the online interface for the dynamic nomogram model.** |

**Reference**

1. Wang X, Sun Y, Zhou X, Shen Z, Zhang H, Xing J, et al. Histogram peritumoral enhanced features on mri arterial phase with extracellular contrast agent can improve prediction of microvascular invasion of hepatocellular carcinoma. Quant Imaging Med Surg. 2022;12(2):1372-84.
2. Jiang H, Wei J, Fu F, Wei H, Qin Y, Duan T, Chen W, et al. Predicting microvascular invasion in hepatocellular carcinoma: A dual-institution study on gadoxetate disodium-enhanced MRI. Liver Int 2022;42:1158-1172.
3. CT/MRI Liver Imaging Reporting and Data System version 2018. American College of Radiology Web site.https://www.acr.org/Clinical-Resources/Reporting-and-Data-Systems/LI-RADS
4. LI-RADS Lexicon (terms and definitions). American College of Radiology Web site.https://www.acr.org/-/media/ACR/Files/RADS/LI-RADS/LIRADS-Lexicon-Table.pdf
5. Segal E, Sirlin CB, Ooi C, Adler AS, Gollub J, Chen X, Chan BK, et al. Decoding global gene expression programs in liver cancer by noninvasive imaging. Nat Biotechnol 2007;25:675-680.
6. An C, Kim DW, Park YN, Chung YE, Rhee H, Kim MJ. Single Hepatocellular Carcinoma: Preoperative MR Imaging to Predict Early Recurrence after Curative Resection. Radiology 2015;276:433-443.
7. Renzulli M, Brocchi S, Cucchetti A, Mazzotti F, Mosconi C, Sportoletti C, Brandi G, et al. Can Current Preoperative Imaging Be Used to Detect Microvascular Invasion of Hepatocellular Carcinoma? Radiology 2016;279:432-442.
8. Ronot M, Chernyak V, Burgoyne A, Chang J, Jiang H, Bashir M, Fowler KJ. Imaging to Predict Prognosis in Hepatocellular Carcinoma: Current and Future Perspectives. Radiology 2023;307:e221429.
9. Lee S, Kim SH, Lee JE, Sinn DH, Park CK. Preoperative gadoxetic acid-enhanced MRI for predicting microvascular invasion in patients with single hepatocellular carcinoma. J Hepatol 2017;67:526-534.
10. Ji GW, Zhu FP, Xu Q, Wang K, Wu MY, Tang WW, Li XC, et al. Radiomic Features at Contrast-enhanced CT Predict Recurrence in Early Stage Hepatocellular Carcinoma: A Multi-Institutional Study. Radiology 2020;294:568-579.
11. Lee S, Kim SS, Bae H, Shin J, Yoon JK, Kim MJ. Application of Liver Imaging Reporting and Data System version 2018 ancillary features to upgrade from LR-4 to LR-5 on gadoxetic acid-enhanced MRI. Eur Radiol. 2021;31(2):855-863.
12. Liang X, Shi S, Gao T. Preoperative gadoxetic acid-enhanced MRI predicts aggressive pathological features in LI-RADS category 5 hepatocellular carcinoma. Clin Radiol. 2022;77(9):708-716.
