# Supplementary material for: Dynamic nomogram integrating Gd-EOB-DTPA enhanced MRI semantic features, nutritional-inflammatory indices, and early treatment response to predict long-term survival in unresectable HCC treated with interventional, targeted, and immunotherapy: a multicenter retrospective study
Source: Front Immunol. 2026 Jul 1;17:1848104. doi: 10.3389/fimmu.2026.1848104 (PMC13368791; doi:10.3389/fimmu.2026.1848104)
Supplement: Supplementary file 2 [file Presentation1.zip › Figure 1.pdf]

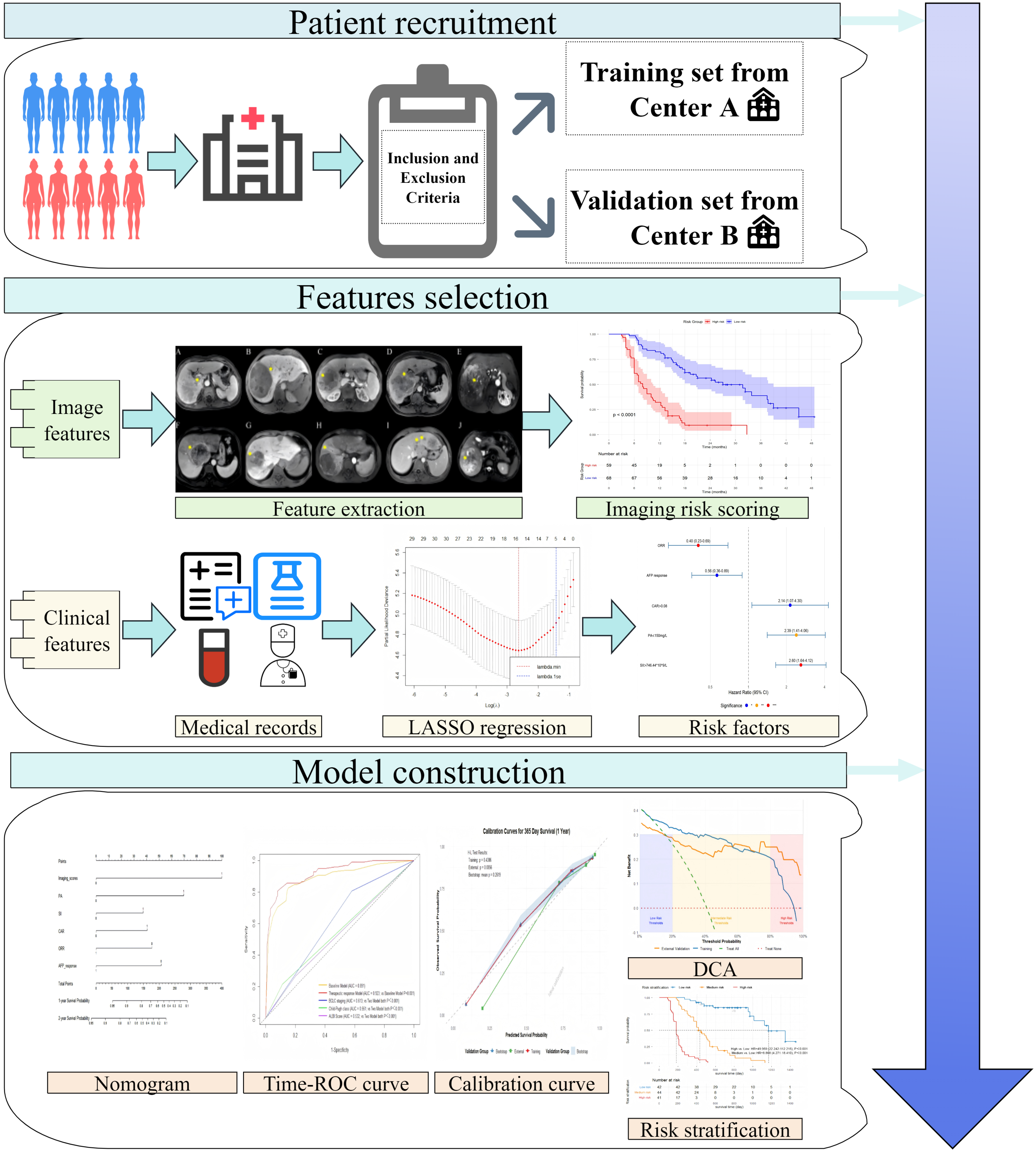
Patient recruitment
Inclusion and Exclusion Criteria
Training set from Center A
Validation set from Center B
Features selection
Image features
Feature extraction
Imaging risk scoring
Clinical features
Medical records
LASSO regression
Risk factors
Model construction
Nomogram
Time-ROC curve
Calibration curve
DCA
Risk stratification
