# Supplementary figures and images for: Dynamic nomogram integrating Gd-EOB-DTPA enhanced MRI semantic features, nutritional-inflammatory indices, and early treatment response to predict long-term survival in unresectable HCC treated with interventional, targeted, and immunotherapy: a multicenter retrospective study
Source: Front Immunol. 2026 Jul 1;17:1848104. doi: 10.3389/fimmu.2026.1848104 (PMC13368791; doi:10.3389/fimmu.2026.1848104)

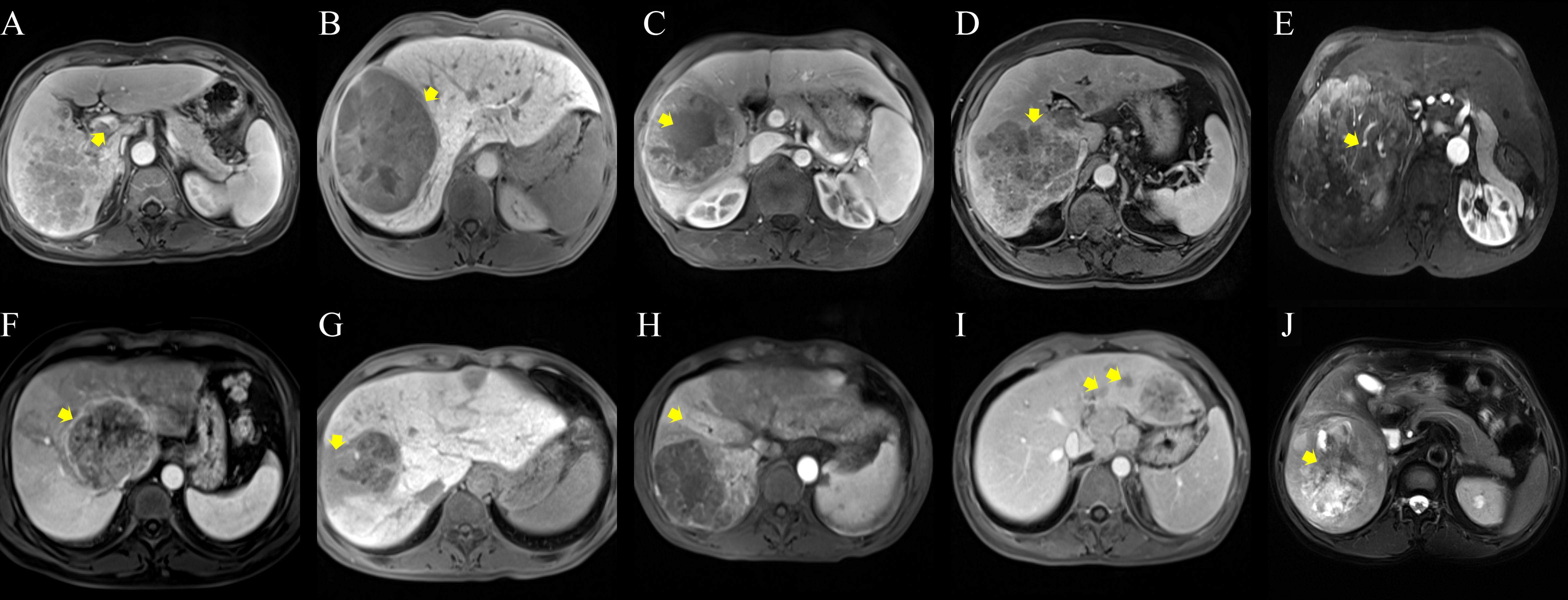

Supplement: Supplementary file 2 [file Presentation1.zip › Figure 2.tif]

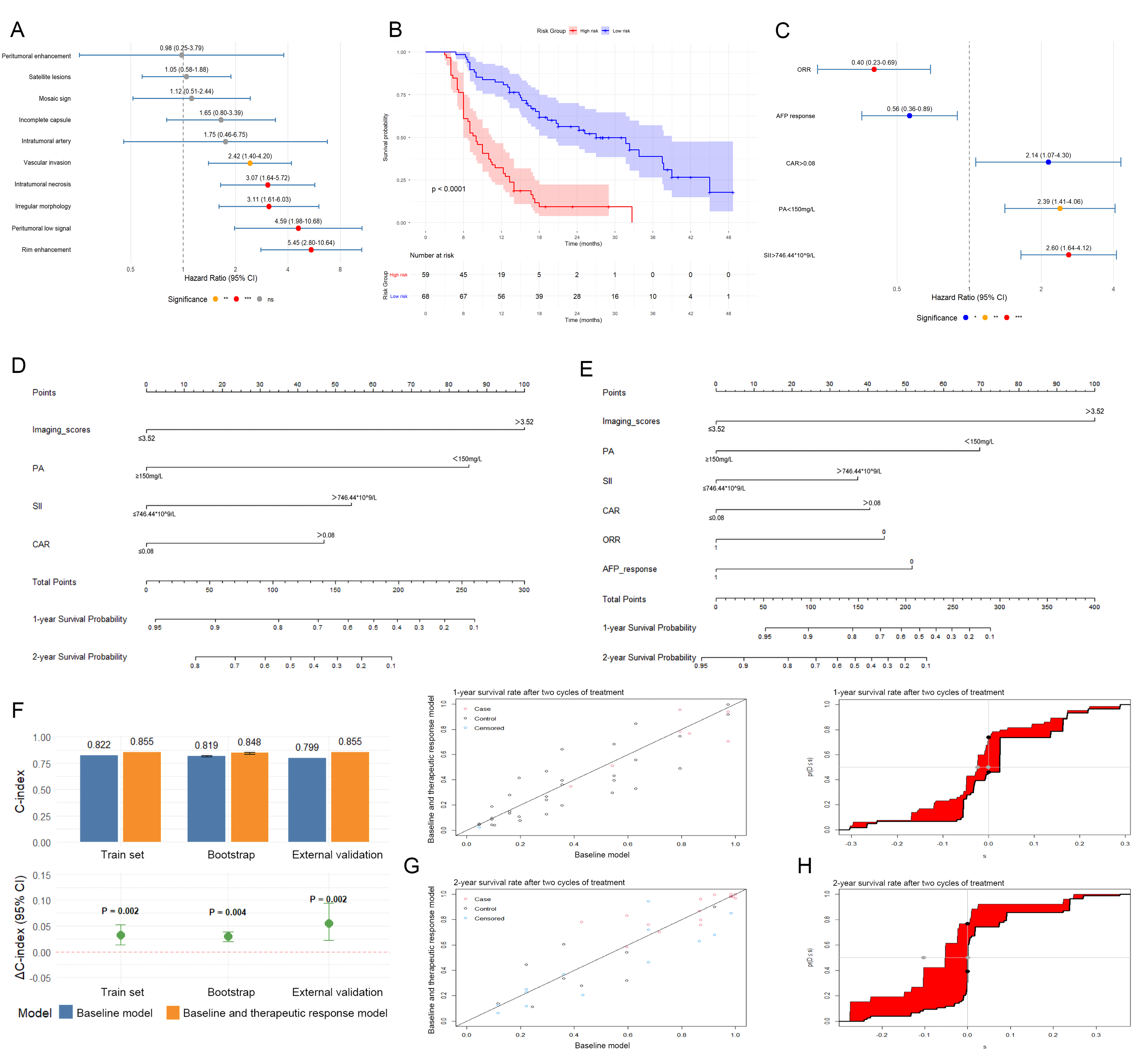

Supplement: Supplementary file 2 [file Presentation1.zip › Figure 3.tif]

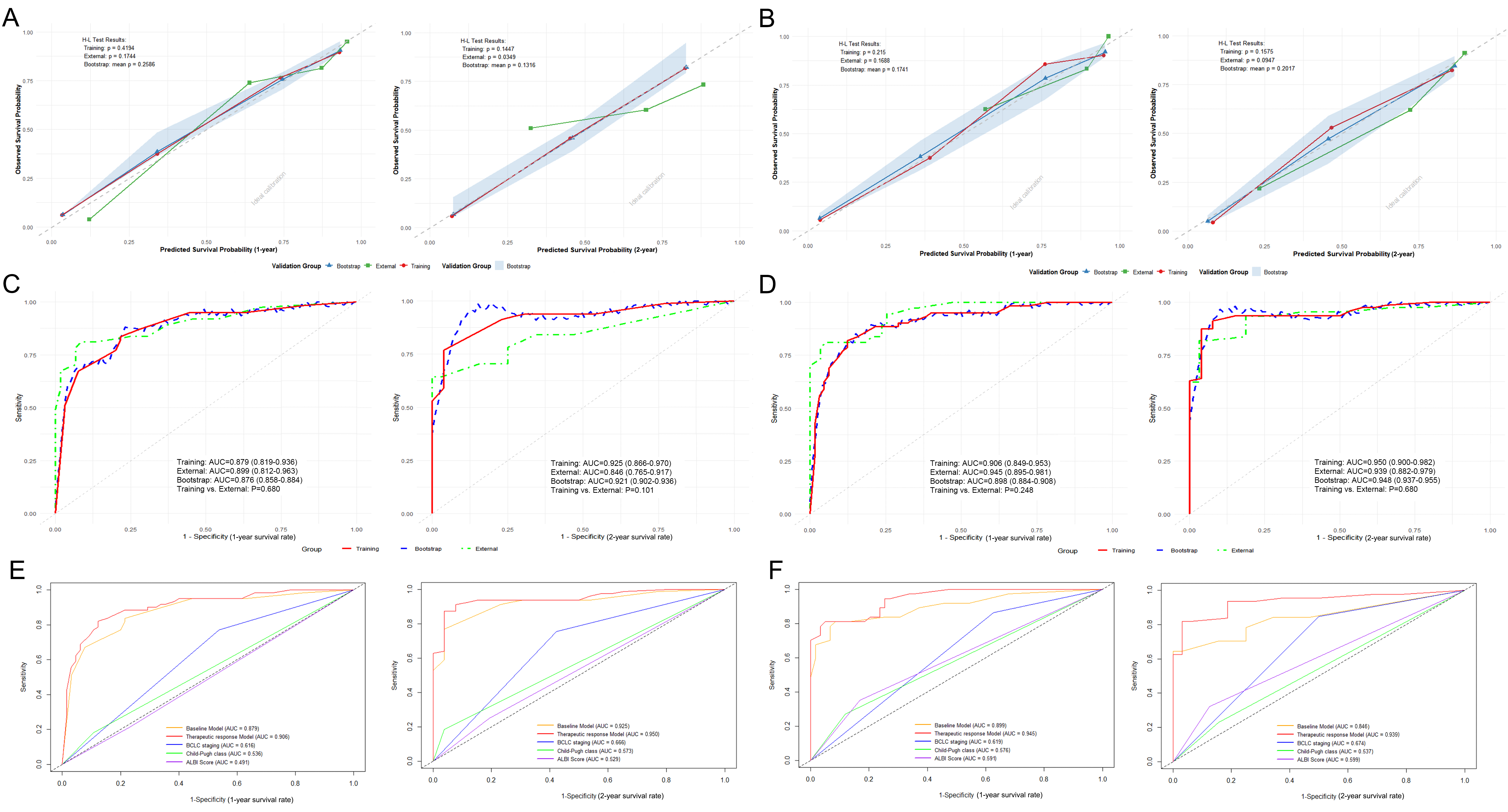

Supplement: Supplementary file 2 [file Presentation1.zip › Figure 4.tif]

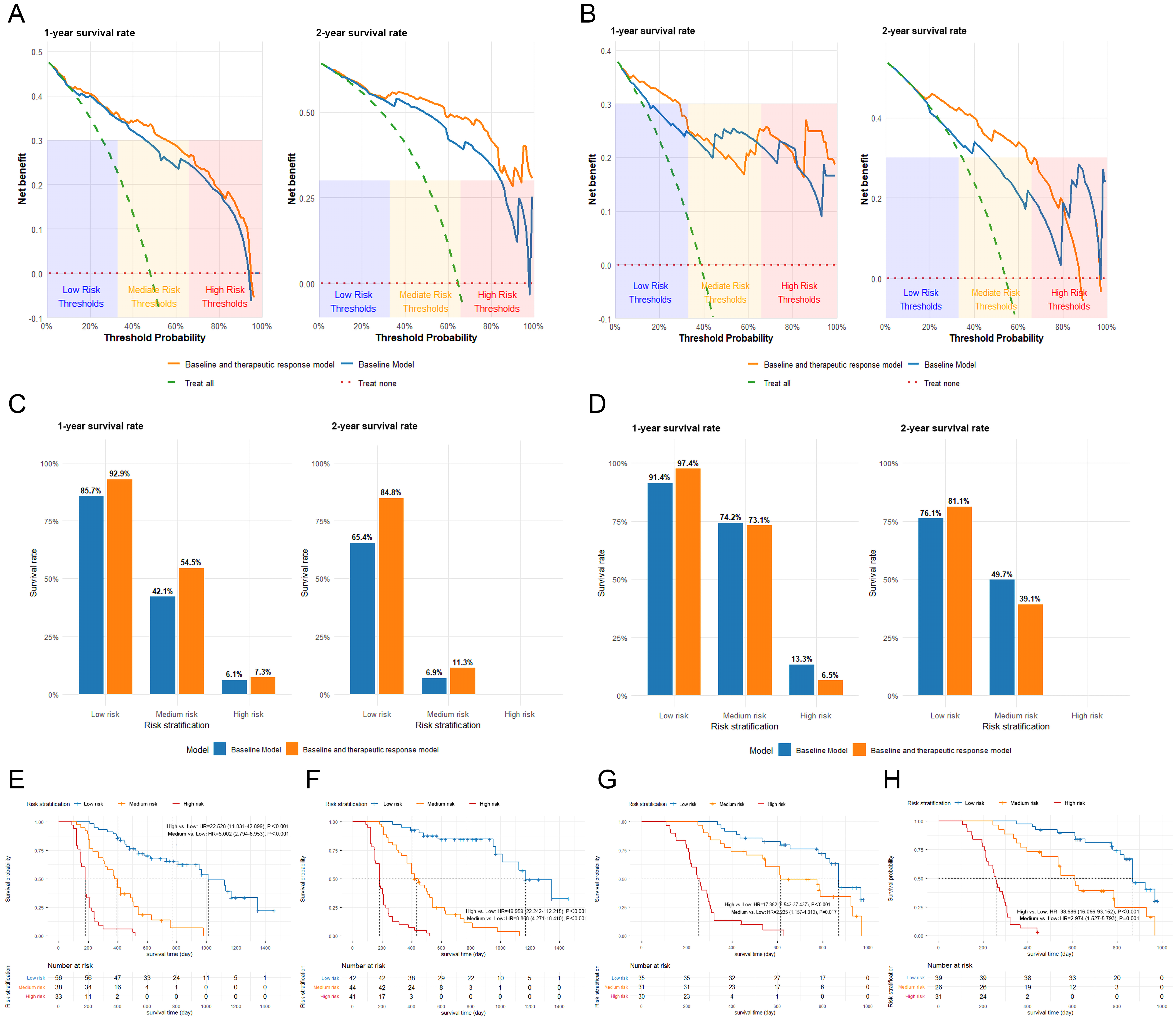

Supplement: Supplementary file 2 [file Presentation1.zip › Figure 5.tif]

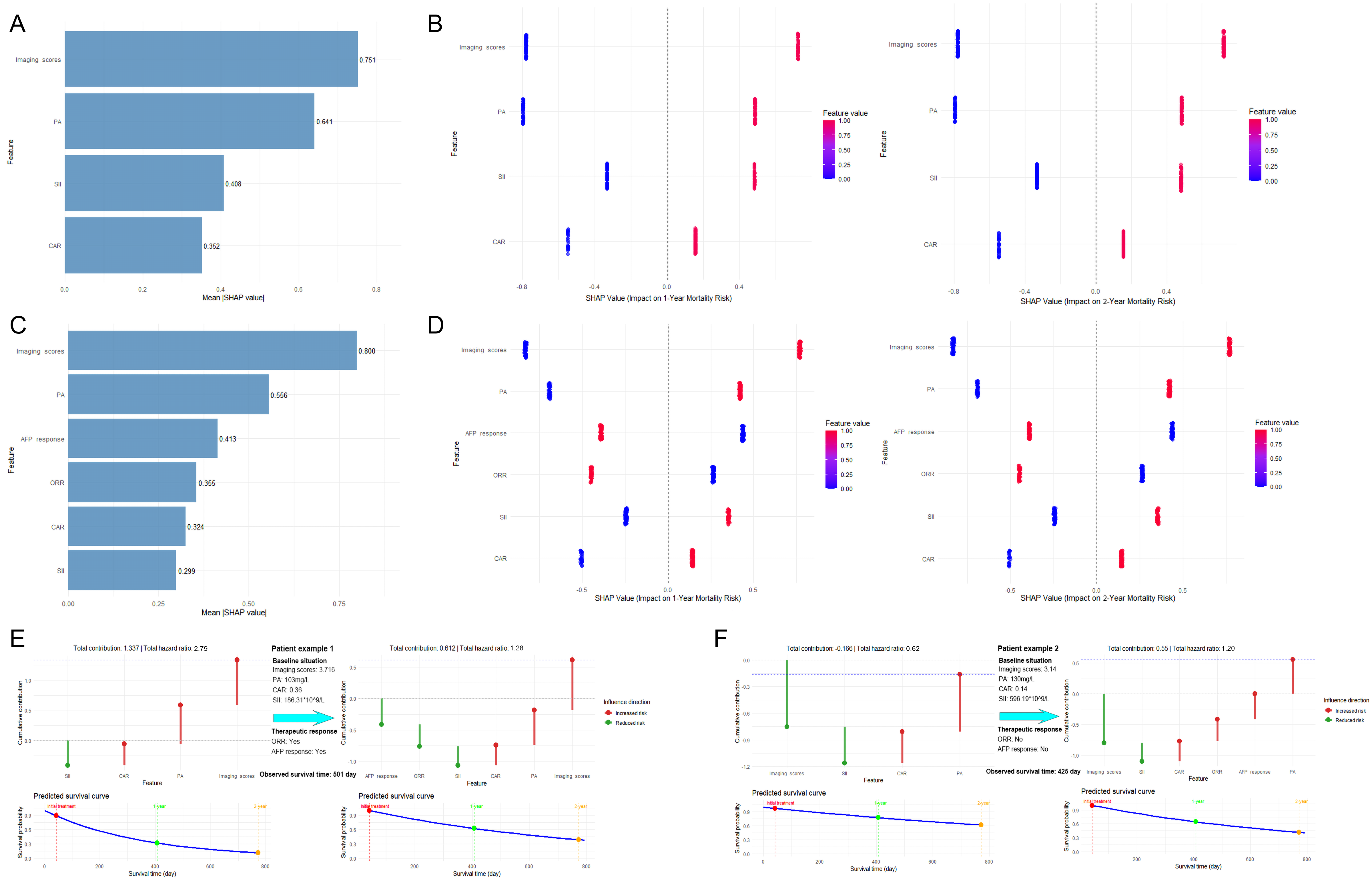

Supplement: Supplementary file 2 [file Presentation1.zip › Figure 6.tif]
